# Organic Nanodelivery Systems as a New Platform in the Management of Breast Cancer: A Comprehensive Review from Preclinical to Clinical Studies

**DOI:** 10.3390/jcm12072648

**Published:** 2023-04-02

**Authors:** Salma T. Rafik, Jayant S. Vaidya, Alexander J. MacRobert, Elnaz Yaghini

**Affiliations:** 1 Division of Surgery and Interventional Science, Faculty of Medical Sciences, University College London (UCL), London W1W 7TY, UK; 2 Department of Clinical Pharmacology, Faculty of Medicine, Alexandria University, Alexandria 21516, Egypt

**Keywords:** breast cancer, nanotechnology, organic nanoparticles, multifunctional, stimuli-responsive, clinical translation, drug chemotherapy, preclinical

## Abstract

Breast cancer accounts for approximately 25% of cancer cases and 16.5% of cancer deaths in women, and the World Health Organization predicts that the number of new cases will increase by almost 70% over the next two decades, mainly due to an ageing population. Effective diagnostic and treatment strategies are, therefore, urgently required for improving cure rates among patients since current therapeutic modalities have many limitations and side effects. Nanomedicine is evolving as a promising approach for cancer management, including breast cancer, and various types of organic and inorganic nanomaterials have been investigated for their role in breast cancer diagnosis and treatment. Following an overview on breast cancer characteristics and pathogenesis and challenges of the current treatment strategies, the therapeutic potential of biocompatible organic-based nanoparticles such as liposomes and polymeric micelles that have been tested in breast cancer models are reviewed. The efficacies of different drug delivery and targeting strategies are documented, ranging from synthetic to cell-derived nanoformulations together with a summary of the interaction of nanoparticles with externally applied energy such as radiotherapy. The clinical translation of nanoformulations for breast cancer treatment is summarized including those undergoing clinical trials.

## 1. Introduction

### 1.1. Breast Cancer Background

**Figure 1:**
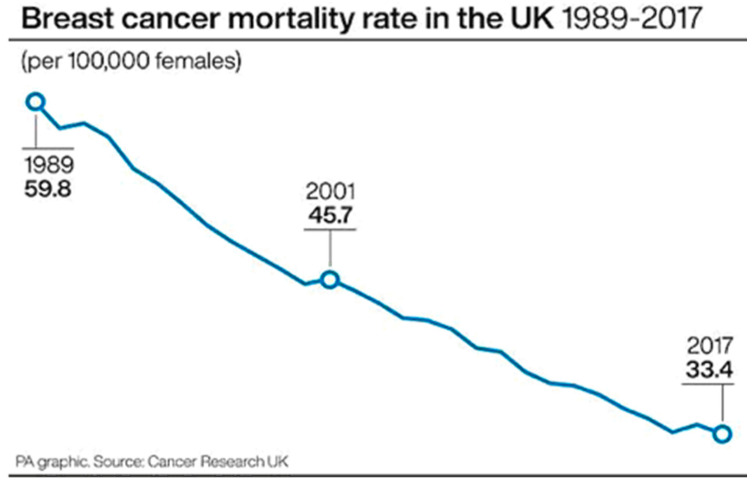
Mortality rate of breast cancer among females in the UK between 1989 and 2017.

In 2020, an estimated 2.3 million new cases of breast cancer and 685,000 deaths were reported worldwide, and breast cancer was first for incidence in 159 countries and for mortality in 110 countries [1]. The World Health Organization (WHO) predicts that the number of new cases will increase by almost 70% over the next two decades, mainly due to an ageing population. Effective diagnostic and treatment strategies are of utmost necessity for improving cure rates among patients. At present, the treatment of breast cancer often comprises a combination of surgical resection, radiation therapy and systemic drug therapy (hormonal therapy, chemotherapy and/or targeted biological therapy). These modalities have managed to greatly reduce population mortality from breast cancer, almost halving it (e.g., from 60/100,000 to 33/100,00 from 1989 to 2017 in the UK, as shown in Figure 1). However, these therapeutic modalities have many limitations and side effects.

The application of nanotechnology in various medical domains has grown enormously in the past decades [1]. In this review, we first discuss the mechanism of breast cancer development, breast tumour heterogeneity and their implications for management strategies. Next, we summarise types of different nanocarrier-based theranostic (i.e., a combination of diagnostic and therapeutic) drug delivery systems based on organic nanoparticles in breast cancer management. Organic-based nanoparticles are likely to be more biocompatible and biodegradable than inorganic-based nanodelivery systems and, therefore, offer greater translational potential, which is underlined by the clinically approved nanodelivery systems for breast cancer. Finally, we provide current perspectives on the potential application of organic-based nanocarriers for management of this cancer.

### 1.2. Breast Cancer Pathogenesis

Enormous effort has been made to characterize the molecular features underlying breast cancer formation and progression. Various possible mechanisms have been proposed for the development of breast cancer, which may occur across multiple phases of life. Carcinogenesis is a result of accumulated damage from both spontaneous and environmentally induced events that contribute to cancer development through multiple stages, beginning with the alteration of cells from a normal healthy state [2]. At the cell-of-origin level, two theories are suggested to be implicated in the process of carcinogenesis, namely the clonal evolution theory and the cancer stem cell theory [3]. The clonal evolution theory first proposed by Nowell in 1976 postulates that cancer cells with various phenotypes arise within a tumour due to accumulating genetic mutations and epigenetic changes, which can then provide a selective growth advantage for the most aggressive cells, thereby driving tumour progression [4]. The cancer stem cell theory suggests that a particular subset of tumour cells with stem cell-like properties, called “cancer stem cells” (CSCs), drive tumour initiation, progression, metastasis and recurrence. These cells have the capability of self-renewal and differentiation, as with normal adult stem cells, which eventually leads to the generation of all cell types of a tumour, thereby accounting for tumour heterogeneity. Cancer stem cells can divide symmetrically to replenish the CSC pool, as well as asymmetrically and irreversibly to differentiate into populations of non-CSCs, which form the bulk of the tumour. A third emerging theory referred to as the “plastic CSC theory” has postulated that the equilibrium of cell states within tumours is maintained through bidirectional cell conversions between “cancer stem cells” (CSCs) and non-CSCs. Cancer stem cells self-renew and form more stem cells, differentiated cells and tumour cells, whereas differentiated tumour cells could dedifferentiate. This theory bridges the two conventional theories, the clonal evolution theory and cancer stem cell theory, and suggests that the presence of plastic cellular populations leads to the development of drug resistance and metastasis [5].

**Figure 2:**
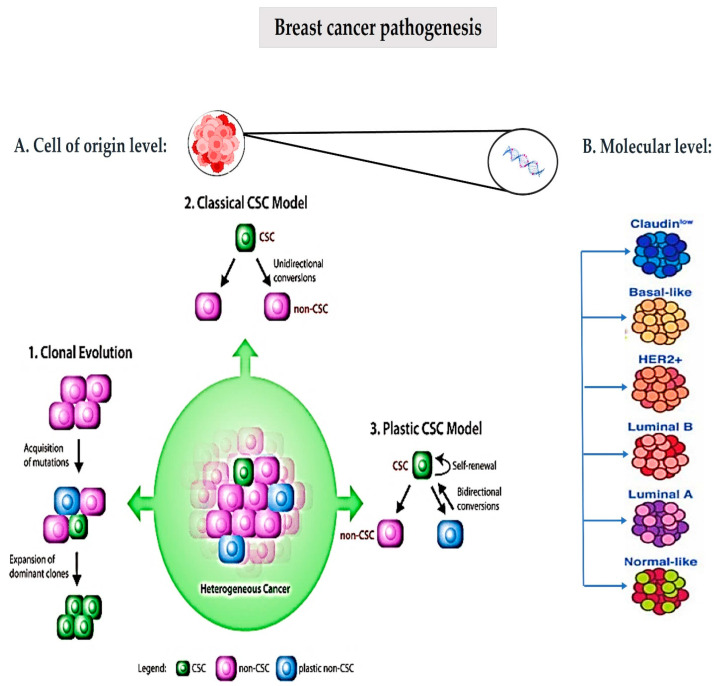
Mechanisms underlying breast cancer pathogenesis. (**A**) Cell-of-origin schematic description of the clonal evolution theory and the classical CSC (cancer stem cell) and plastic stem cell models; (**B**) Molecular studies of numerous genetic and epigenetic alterations has led to the classification into six subtypes (normal-like, luminal A, luminal B, HER2-enriched, claudin-low, and basal-like).

There is ample evidence showing that numerous genetic and epigenetic alterations trigger breast tumourigenesis. Gene expression studies have distinguished several breast cancer subtypes related to oestrogen receptor (ER) expression (the luminal cluster), human epidermal growth factor 2 (HER2) expression and the basal cluster of genes. Breast cancer is, thus, divided into six subtypes (normal-like, luminal A, luminal B, HER2-enriched, claudin-low and basal-like), with differing clinical prognoses and responses to therapy [6], as depicted in Figure 2. Luminal A cancers generally have the best prognosis followed by normal-like cancers. HER2-positive and basal-like breast cancer are fast-growing and have a worse prognosis. Triple-negative (negative oestrogen, progesterone and HER2 receptor expression) basal-like cancer is the most aggressive and difficult to treat amongst breast cancers, and occurs more frequently in women with BRCA1 gene mutations. Claudin-low is a more recently described class, largely triple-negative but differs in that it has low expression of cell–cell junction proteins, including E-cadherin, which increases metastatic potential and frequent infiltration with lymphocytes [7].

## 2. Breast Tumour Heterogeneity: Implications for Management Strategies

Breast cancer can be highly heterogeneous, with tumours showing variable morphologic, phenotypic and biological features. These variations can evolve with time and in different regions of the tumour, and are categorised as interpatient heterogeneity (variations between tumours in different patients), intertumoural (variability between primary tumour and metastases) and intratumoural heterogeneity (diversity within different regions of the same tumour mass). Such heterogeneity makes the study and treatment of breast cancer difficult because tumour samples (taken as needle core biopsy) may not be representative of the whole lesion [8], although overall, the behaviour and treatment response predictions based on core biopsy are fairly accurate. The molecular basis for inter and intratumoural heterogeneity comprises two main parameters: firstly, cancer-cell-intrinsic factors, for instance, genetic alterations, interaction between the genome, epigenome/transcriptome, proteome, proliferation, stemness and intrinsic cell plasticity, invasion and metastatic capabilities; and secondly, microenvironmental factors such as tumour hypoxia, the degree of vascularisation, variability in tumour pH and interactions of cancer cells with surrounding stromal cells (e.g., endothelial cells, fibroblasts, pericytes, adipocytes and variable tumour-infiltrating cells of the innate and the adaptive immune systems) [9]. The growing tumour mass creates internal distortion, for example, constriction of blood vessels, and external distortion upon adjacent normal tissue, which, in turn, exerts reciprocal mechanical stress on the tumour. Further factors that contribute to the internal stress and stiffness of the extracellular matrix (ECM) are the growth of tumour-associated fibroblasts and secretion of ECM components, chiefly collagen [10].

Another type of tumour heterogeneity that is seldom taken into consideration but is significant from a therapeutic perspective, mainly for metastatic deposits, is the pharmacokinetic and pharmacodynamic aspects related to tumour heterogeneity, and even small drugs exhibit heterogeneous distribution in different regions of a tumour [11,12,13] and are almost absent in some parts [14]. Histological examinations show that blood vessels are mostly collapsed near the core of the tumour and distorted elliptically towards the periphery [15], which hinders uniform drug delivery throughout the tumour, with higher levels accumulating near the periphery. In addition, the higher interstitial fluid pressure within the tumour further hinders the access of the drug to the core [16]. Overall, blood supply is most likely the determining factor between tumour size and drug levels, as larger tumours often have hypoperfused regions, whereas small tumours have more consistent vascularisation [10]. Intratumoural heterogeneity also reflects the tumour’s adaptability through phenotypic selection in response to hypoxia or exposure to chemotherapy or other insults, which can then confer treatment resistance to the tumour cells. Hence, the heterogeneous nature of breast cancer represents a major challenge for diagnosis and therapy, which has driven research into the molecular-based stratification of patients to reach a comprehensive diagnosis with the aim of selecting the right therapeutic strategy and identifying genetic changes that drive treatment resistance as well as therapy adjustments [6,17].

## 3. Challenges of Conventional Strategies for Management of Breast Cancer

Current therapeutic approaches comprise local and systemic treatments, which can be used in combination or alone. The primary treatment, if the cancer has not metastasised, is surgery, to which adjuvant radiotherapy may be added. Systemic treatments include chemotherapy, immunotherapy, targeted and hormone therapy. In patients with early-stage tumours, the mainstay line of treatment is local therapy, with surgical removal of the tumour and surgical assessment of the axilla, and, if necessary, removal of axillary lymph nodes. When the breast is preserved, radiotherapy is given either during the operation (TARGIT-IORT) [18,19] or as a post-operative course for several weeks. Adjuvant systemic treatments are important after local treatment in a proportion of patients to reduce the risk of relapse and death. This is important because once the cancer progresses and spreads to other organs, the role of local treatments becomes less important and can be even detrimental. Accordingly, systemic drug therapy then becomes the mainstay for controlling advanced breast cancer.

Hormonal therapy uses drugs that affect how hormones stimulate the growth of cancerous cells [20]. Oestrogen has been well recognized as a key driver in breast cancer progression. Hormonal therapy such as selective oestrogen receptor modulators, aromatase inhibitors and gonadotropin-releasing hormone analogue are used in oestrogen-dependent breast cancers through the inhibition of the oestrogen signalling pathway or blocking the biosynthesis of androgens by inhibition of aromatase enzyme, resulting in a reduction in oestrogen levels or inhibition of oestrogen production, respectively. Various groups of conventional chemotherapy are used for the treatment of breast cancer by killing tumour cells via multiple mechanisms. Among the most commonly used are anthracycline drugs such as doxorubicin, taxane drugs such as paclitaxel and docetaxel, platinum drugs, cyclophosphamide and others. A major drawback in using conventional chemotherapy is systemic toxicity, which has prompted the development of targeting particular molecules related to tumour cells so as reduce damage to normal cells. Trastuzumab (i.e., Herceptin) is a well-known targeted therapy that uses a humanized anti-HER2 monoclonal antibody for reducing the risk of cancer relapse. Another monoclonal antibody that was tested as a targeted therapy of breast cancer (but with poorer results) is bevacizumab, which recognizes vascular endothelial growth factor (VEGF) [21]. Immunotherapy is another approach that involves stimulation of the immune system, for example, through the use of checkpoint inhibitors that aid and restore the functions of the immune system to fight against tumour cells [20]. Despite these advances in the management of breast cancer, it is still not possible to cure patients at metastatic stages of the disease.

Numerous obstacles also account for the limitations of conventional radiation-based therapies in breast cancer. Radiotherapy can have toxic side effects such as dose heterogeneity, local discomfort and long-term exposure to healthy tissues. Many of the side effects are potentially reversible and self-limiting, but some can be irreversible and progressively worsen. Cardiac toxicity, lymphedema and pneumonitis are amongst the known side effects of radiotherapy for breast cancer, although some of these can be avoided through the use of intraoperative radiotherapy [18,19,22]. However, the development of radio-resistance remains a confounding factor since higher radiation may become necessary, thereby increasing the risk of side effects [23].

The obstacles to the success of systemic drug therapy arise for pharmaceutical, pharmacokinetic and pharmacodynamic reasons. Major limiting factors to anticancer drug delivery include low aqueous solubility, poor in vivo stability and toxicity of the drugs or the formulation agents, such as surfactants and organic co-solvents. Pharmacokinetic obstacles that hinder reaching optimum drug concentration at the target tumour site include the rapid clearance and short half-life of drug molecules. Alteration in drug cellular uptake processes by cancer cells is another issue. It has been proposed that cancer cells develop multiple mechanisms that eventually impair drug uptake by the cells, for example, changes in lipid plasma membrane composition, downregulation of the expression of drug transporters that promote drug uptake by the cell and overexpression of efflux pumps that expel drugs out of the cells such as ATP-binding cassette pumps (e.g., P-glycoprotein, P-gp) [24]. Finally, there are pharmacodynamic hurdles that reduce the efficacy of a drug and lead to the development of drug resistance, such as structural alterations of the drug targets, and the presence of compensatory signalling pathways that can bypass the mechanism of drug action. As previously mentioned, at the beginning of treatment, there may be a small number of tumour cells harbouring mutations that are resistant to the applied cancer treatment as a result of genomic variance. The cytotoxic treatments may target and effectively kill one tumour clone; however, surviving cancer cells continue to evolve and may develop resistance to treatment. Genomic instability may also contribute to the ability of cancers to become drug-resistant, whereby the accumulation of new somatic mutations can confer a survival advantage in that the treatment cannot effectively kill or slow the progression of this clone variant [24]. In summary, many challenges need to be overcome in order to improve breast cancer treatment.

The introduction of nanotechnology to oncological research through the development of nano-based drug delivery systems and imaging agents has made a major impact as part of a field more widely known as “nanomedicine” [25]. Nanoparticles (NPs) are generally defined as particles with a mean diameter in the range 1–100 nm. Although not commonly used in clinical treatments to date, NPs have the potential to address some of the challenges encountered by the conventional breast cancer therapies, as summarised in the following sections.

## 4. Breast Cancer in the Era of Nanomedicine

Investigation of the use of nanoparticles in cancer treatment has been driven by several advantageous factors. Firstly, the solubility, stability and half-life of drugs can be improved by incorporation or attachment of the drug to the nanoparticle, thereby increasing the bioavailability of many chemotherapeutic agents. If the drug is prone to hydrolysis, for example, then its incorporation within a nanoparticle matrix can improve its stability. Secondly, the use of NPs can increase the drug accumulation in cancer tissues either via passive targeting through the enhanced permeability and retention (EPR), or active targeting by using various ligands targeted to overexpressed tumour antigens. Accordingly, NPs can enhance tumour specificity, and reduce drug levels within normal tissues and adverse side effects. In contrast to small drugs, the uptake of NPs by cells is mainly via endocytosis, which can counteract cellular drug efflux mechanisms. The versatility of nanoparticles is illustrated in their range of applications and functions. Several agents may be co-delivered in NPs for combination therapy, or where one agent is used for imaging, combined imaging and therapy. Furthermore, it is possible to control the rates of drug release from the NPs both spatially and temporally using stimuli-responsive nanoformulations. Moreover, NPs offer the possibility of co-delivery of agents that target drug resistance mechanisms (tumour microenvironment, cancer stem cells, etc.) [25]. Nanoparticles can also be used to deliver biomolecules such as siRNA, mRNA and miRNA to their sites of action intracellularly and facilitate gene therapy for cancer treatment [26].

As well as chemotherapy, nanotechnology has substantial applications in radiotherapy, which include enhancing the delivery of radioisotopes to tumour tissues with higher specificity through passive or active targeting and by combining chemotherapy and radiotherapy in the treatment of breast cancer. In this scenario, the chemotherapy sensitises the tumour cells to radiotherapy through the radiosensitising capabilities of common chemotherapeutics such as cisplatin, doxorubicin and paclitaxel [27]. Such approaches illustrate the advances being made in the design of multifunctional nanodelivery systems according to the diagnostic and/or therapeutic objectives [28].

Nevertheless, there remain concerns about retention of nanoparticles within the body, their clearance rate and potential toxicity, which may hamper clinical translation and regulatory approval in some cases. Nanoparticles can be prepared in different sizes, shapes and ranges of stability and drug loading capacity, but can be classified structurally into two main categories based on their composition, namely ‘organic’ and ‘inorganic’. Organic-based nanoparticles mainly encompass lipid-based and polymer-based NPs, including dendrimers. Inorganic nanoparticles include metal-based (e.g., gold), quantum dots and silica nanoparticles [29]. In this review, we have focused on organic nanoparticles that can be degraded under physiological conditions, since the faster clearance and excretion rate of the smaller biodegradation products should favour short-term retention, thereby minimising toxicity and facilitating regulatory approval. In addition to the biocompatibility of organic-based nanoparticles, biodegradability can be utilised for controlling drug release at the target site, thereby limiting off-target effects.

**Figure 3:**
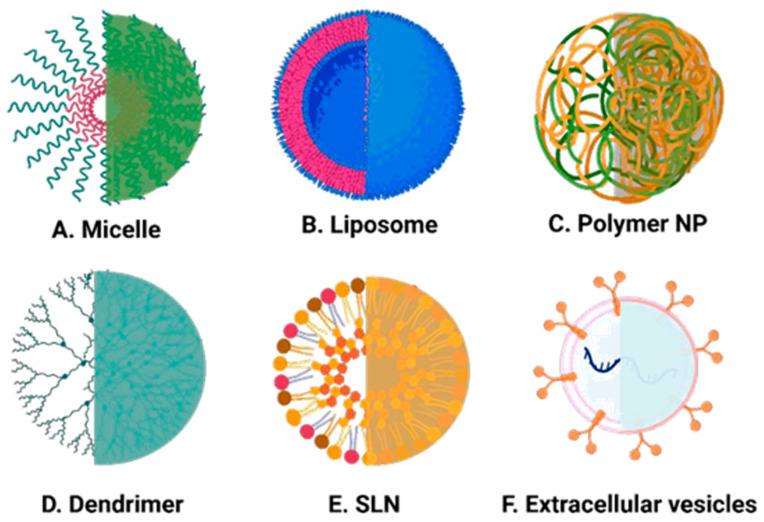
Schematic presentation of the cross-sectional structure of classes of organic nanoparticles. (**A**) Micelle with hydrophobic component of the polymeric surfactant within the interior where hydrophobic drugs can be incorporated; (**B**) Liposome with lipid bilayer and aqueous core; (**C**) Polymer nanoparticle composed of a matrix of polymeric components; (**D**) Dendrimer showing symmetric branched polymeric structure; (**E**) Solid lipid nanoparticle (SLN) with surfactant surface and lipid interior; (**F**) Extracellular vesicles showing an example of a cell-derived membrane-bound vesicle (exosome) incorporating a drug in the interior.

We have summarised the various types of organic-based nanoparticles that have been investigated for application in breast cancer treatment in the following section. We begin with lipid-based nanoparticles, and Table 1 lists recent studies on lipid-based nanoformulations and their application in experimental studies of breast cancer. The experimental model employed in each case is described, together with the experimental outcome. In Table 2, we have listed details of polymer-based nanoformulations, including hybrid lipid–polymer formulations. Figure 3 shows schematic structures of the main classes of organic nanoparticles.

### 4.1. Lipid-Based Nanoparticles

Among the many nanoformulations that have been investigated for the treatment of cancer in research, lipid-based nanoformulations are the most studied. There are several different types of lipid-based formulations such as liposomes, solid lipid nanoparticles (SLN) and nanostructured lipid carriers (NLC), which are discussed in the following sections.

#### 4.1.1. Liposomes

Liposomes are spherical vesicles that can be divided into the categories of small unilamellar vesicles with a single lipid bilayer approximately 100 nm in size, and larger unilamellar and multilamellar vesicles containing multiple bilayers with sizes that can range to several hundred nm. The bilayer is typically composed of amphipathic phospholipid molecules, which enables suspension and storage in aqueous solution. Liposomes are versatile and can be used to deliver a wide range of agents of differing water solubility either within the central aqueous compartment or the lipid bilayer in the case of hydrophobic drugs, whilst conferring protection against degradation in the circulation [75]. Owing to the similarities in the phospholipid composition to that of cell membranes, liposomes also offer superior biocompatibility than many synthetic materials. They have a range of other favourable properties: i.e., non-haemolytic, non-immunogenic, non-toxic and biodegradable. The exterior of liposomes are often modified by attaching polyethylene glycol chains (PEGylation) in order to protect the surface of the liposomes from serum protein binding and prolong their circulation time. A notable example is Doxil, as discussed later in the clinical section, which is a PEGylated liposomal formation with doxorubicin encapsulated within the internal aqueous compartment, and was the first ‘nano-drug’ to be approved by the FDA. The targeting of liposomes can also be achieved through surface functionalisation with targeting ligands [75]. In addition to liposomes, several novel vesicular nanocarriers such as niosomes and transferrosomes that resemble liposomes in structure, and are capable of transporting both hydrophilic and hydrophobic agents, have recently been developed, but no clinical data are available [76].

Among the most frequently used chemotherapy regimens in the treatment of breast cancer are those that encompass anthracyclines, specifically doxorubicin and epirubicin (FDA-approved), and/or taxanes, specifically docetaxel and paclitaxel (FDA-approved), although in selected patients, cyclophosphamide/methotrexate/5-fluorouracil (CMF) may still be used. The addition of taxanes slightly improves the efficacy of chemotherapy, but at the expense of increased non-cardiac toxicity; most importantly, it allows the use of a lower total dose of anthracyclines by using sequential regimens. Chemotherapy regimens based on anthracyclines and taxanes are effective in reducing mortality among patients with breast cancer, but potential long-term sequelae of induced cardiotoxicity are of particular concern. As a result, a study by Franco et al. found that compared to free paclitaxel and doxorubicin, a 1:10 co-encapsulation ratio of paclitaxel and doxorubicin in liposomes decreased the cardiac toxicity profile in mice bearing 4T1 breast tumour [77]. Papa et al. (2013) assessed the cytotoxic effect of gemcitabine-loaded liposomes in vitro and in vivo (4T1 breast tumour-bearing mice) using different breast cancer cell lines. Gemcitabine-loaded liposomes showed significant cytotoxicity at lower doses compared to equivalent does of the free drug against the MDA-MB-231 and 4T1 cell lines at 48 and 72 h [30]. In a similar study by Coscoa et al., tamoxifen (a lipophilic drug) and gemcitabine (a hydrophilic drug) were loaded in liposomes (mean size of 150–200 nm), and the in vitro antitumoural activity was tested on different breast cancer cell lines (MCF-7 and T47D cells). Both drugs demonstrated a synergistic effect, and tamoxifen was found to modulate the release of gemcitabine. In addition, liposomal formulations induced a higher reduction in cell viability compared to the free drug forms, while the liposomal gemcitabine-tamoxifen co-encapsulated formulation had the best antitumoural action, particularly after 48 and 72 h incubation time [78]. Another study carried out by Wong and Chiu investigated co-encapsulated vincristine and quercetin PEGlyated liposomes for the treatment of hormone- and trastuzumab-insensitive breast cancer. This study showed that using a liposome to encapsulate both drugs resulted in a prolonged drug circulation time with controlled drug release and higher synergism in vivo for JIMT-1 cells (a trastuzumab-resistant cell line) compared to the two individual drugs [79]. Zhao et al. synthesized liposomes with propylene glycol and trehalose to attain better stability and drug release. This study tested epirubicin-loaded liposomes on MDA-MB 435 cells and their resistant (MDA-MB 435/ADR) counterpart. In vitro experiments revealed higher uptake of the liposomal formulation of epirubicin in the nucleus of tumour cells via endocytosis, and in vivo studies showed significant inhibition of the growth of tumours grown from both MDA-MB-435 ADR lines versus free epirubicin [31].

#### 4.1.2. Solid Lipid Nanoparticles

Solid lipid nanoparticles (SLNs) usually have a spherical shape and diameter size in the range of 50–1000 nm. They are composed of a solid lipid core matrix and stabilized by a surfactant coating. SLNs offer high drug loading, efficient drug release and high long-term stability (at room temperature and at human body temperature) and ease of scale-up for mass production [80]. For example, camptothecin-loaded SLNs elicited increased efficacy against MCF-7 cells and the ‘normal’ MCF 10-A counterpart. Cytotoxicity, uptake and SLN retention were higher compared to the free drug [81]. In another study, docetaxel-loaded SLNs were found to reduce the side effects of docetaxel, particularly myelosuppression, and suppress breast cancer effectively both in vitro and in a mice model with human xenograft breast cancer [82]. A study by da Rocha et al. explored the effect of docetaxel-loaded solid lipid nanoparticles in a 4T1 murine breast cancer cell line and in tumour-bearing mice. The drug-loaded nanoparticles had high docetaxel entrapment efficiency (86%) and exhibited a controlled drug release profile. In vitro studies showed that the IC50 of SLN-DTX against 4T1 cells was more than 100 times lower than that of free DTX after 24 h treatment. In vivo studies showed that SLN-DTX displayed higher antitumour efficacy, as evidenced by reducing tumour volume, and also prevented spontaneous lung metastasis in 4T1 tumour-bearing mice compared to free docetaxel [83]. In a study by Wang et al., the synthesized formulation of resveratrol solid lipid nanoparticles was tested in vitro and in vivo. The results showed that SK-BR-3/PR cells treated with Res-SLNs displayed significant inhibition of cell migration and invasion compared with free resveratrol. Moreover, tests in SK-BR-3/PR xenograft tumour models showed that Res-SLNs had greater efficacy in promoting apoptosis of tumour cells when compared to free resveratrol [84]. Xu et al. prepared paclitaxel-loaded solid lipid nanoparticles for increasing drug uptake in MCF-7 cells. The nanoparticle formulation showed a significantly higher efficacy against the resistant MCF-7/ADR counterpart versus free paclitaxel due to the circumvention of cellular efflux pumps via endocytosis of the SLN [42].

#### 4.1.3. Nanostructured Lipid Carriers

Nevertheless, SLNs exhibit some disadvantages as drug delivery agents, for example, a tendency for aggregation and gelation, polymorphic transition and, in certain cases, low incorporation because of the crystalline structure of the solid lipids. As a result, nanostructured lipid carriers (NLCs) were developed to address some of these drawbacks, and consist of a combination of various lipids, i.e., a solid lipid matrix incorporating a liquid lipid. Adjusting the ratio of solid to liquid lipid can enhance the solubility of drugs, thus improving the encapsulation efficiency and the rate of drug release [85,86]. For example, a study by Di et al. examined doxorubicin and cisplatin co-encapsulated NLCs for breast cancer treatment. The formulated nanocarriers were tested in vitro on MCF-7 cells and MCF-7/ADR cell lines and in vivo on MCF-7/ADR tumour-bearing mice. The results revealed that the NLC nanocarriers co-encapsulating both drugs exhibited the highest antitumour activity, with significantly improved efficiency versus free drug solutions [87]. Furthermore, Marcial et al. demonstrated that paclitaxel encapsulated in nanostructured lipid carriers (75 nm diameter) was highly effective against MCF-7 (IC50 = 25.3 ± 3.2 nM) and MDA-MB-231 cell lines (IC50 = 2.1 ± 0.2 nM), compared to free paclitaxel (IC50 > 500 nM) [88]. In another study by Makeen et al., nanostructured lipid carriers loaded with imatinib were prepared and examined for their in vitro efficacy in MCF-7 cells. All of the NLCs showed slow and sustained release behaviour, and cytotoxicity studies revealed an 8.75-time increase in cytotoxicity (IC50 = 6 µM) vs. imatinib alone (IC50 = 52.5 µM) [89].

Sun et al., 2014, developed quercetin-loaded nanostructured lipid carriers in an attempt to overcome some well-known limitations of quercetin, such as low aqueous solubility and poor bioavailability. The entrapment efficiency reached 95%, and the extended-release of quercetin facilitated MCF-7 and MDA-MB-231 cell killing [46]. Another study by Fernandes et al. investigated the antitumour activity of nanostructured lipid carriers loaded with αtocopherol succinate (TS) and doxorubicin (DOX), which elicited a slow release profile for doxorubicin to free DOX and superior antitumour activity in the 4TI xenograft model [90].

### 4.2. Polymer-Based Nanoparticles

#### 4.2.1. Polymeric Nanoparticles (PNPs)

PNPs are colloidal particles typically with a size of a few hundred nanometres, formulated from biodegradable natural sources such as cellulose, chitosan and Poly(L-lysine) (PLL), which is the polymerized form of lysine, or they can be synthetic polymers such as poly(caprolactone), poly (glycolic acid), polylactic acid (PLA) and Poly(lactide-co-glycolide) (PLGA). PNPs are suitable for carrying hydrophilic and hydrophobic drugs and are prepared by a variety of methods. The anti-cancer drug can be loaded onto the surface of polymeric nanoparticles through surface adsorption, chemical conjugation or encapsulation into the polymeric nanoparticle drug delivery systems. Depending on the method of drug entrapment, polymeric nanoparticles are divided into nanospheres and nanocapsules. Biodegradable polymers offer numerous benefits, for example, better drug encapsulation, higher solubility and permeability and controlled release property of chemotherapeutics agents, which takes place through degradation of the polymeric membrane. They also show remarkable biocompatibility since they are broken down into readily metabolizable polysaccharides in the body as a result of enzymatic degradation [91]. PLGA is an attractive delivery system due to its high stability and low toxicity, as well as a tuneable degradation rate according to the ratio of lactic acid and glycolic acid. Polycaprolactone (PCL) is a hydrophobic semicrystalline polymer that is characterized by having a high drug-binding capacity and biodegradable properties since its ester bonds can degrade under physiological conditions. In addition, PCL shows facile endocytosis-mediated cellular uptake, low toxicity and sustained drug release. Cyclodextrin-derived polymeric nanoparticles are another example of synthetic polymeric nanoparticles. They are water-soluble synthetic carbohydrates that comprise six to eight glucose units in a ring structure. Cyclodextrins generate an amphiphilic cup shape structure with a hydrophilic exterior and hydrophobic interior [92]. For example, in a study carried out by Ya-Jing Ye et al., a hydrophilic cyclodextrin derivative was used to deliver paclitaxel by loading it into the hydrophobic cavity of the cyclodextrin. The resulting complex thereby solubilised PTX, and the complex could then be incorporated into chitosan (CS) nanoparticles. Pharmacokinetic studies in comparison to Taxol^®^ showed that the complex-loaded chitosan nanoparticles exhibited sustained release of the PTX [93]. In another study, DeVeaux et al. synthesized chitosan/polylactide (PLA) nanoparticles loaded with tamoxifen for the treatment of triple-negative breast cancer. The formulation elicited high encapsulation, sustained release and effective killing in vitro [94]. Tran et al. formulated docetaxel (DTX)-loaded PLGA nanoparticles and evaluated their efficacy for DTX delivery to MCF-7 and MDA-MB-231 cells. They showed that treatment with the nanoparticles succeeded in lowering the IC50 values 3.9- and 6.7-fold versus the free drug [95]. Guixia Ling et al. showed that the incorporation of negatively charged dextran sulphate into PLGA nanoparticles (DPNs) led to efficient encapsulation of vincristine (VCR) at 93.6%. In vitro drug release studies at pH 7.4 showed >80% release from VCR-DPNs after 96 h, and pharmacokinetic studies in rats using an oral bolus elicited superior bioavailability of VCR-DPNs versus free VCR. Cell uptake studies in sensitive MCF-7 and resistant P-glycoprotein-overexpressing MCF-7/Adr cells demonstrated 12-fold higher uptake in the resistant cell line versus free VCR, showing that P-glycoprotein-mediated drug efflux was abrogated by the use of DPN [96]. Xiong et al. investigated the antitumour effect of polyethylene glycol/ε-caprolactone (PCEC) nanoparticles for the co-delivery of paclitaxel and curcumin in vitro and in vivo. The NPs exhibited a dose-dependent cytotoxicity in MCF-7 cells, with a higher apoptosis rate and cell uptake compared to the free drugs. Intravenous administration of the nanoparticles to murine MCF-7 xenografts resulted in significant tumour growth inhibition and prolonged survival time, with minimal side effects when compared with free drugs. Furthermore, a lower Ki67 expression (*p* < 0.05) and higher apoptosis was found in tumour cells compared to other treatment groups [97]. In another study by Elahe Akbari et al., the synergistic anticancer efficacy of trapoxin A (TPX) and methotrexate (MTX) co-loaded PLGA-PEG nanoparticles against MCF-7 cells was investigated. The TPX/MTX co-loaded nanoparticles exhibited greater growth inhibition against MCF-7 cells compared to the free drugs [54].

#### 4.2.2. Polymeric Micelles

This drug delivery system is composed of amphiphilic block copolymers that self-assemble into a core–shell micellar structure. The hydrophobic core is intended for water-insoluble drugs, while the hydrophilic shell is intended for hydrophilic drug loading. In addition, core cross-linked polymer micelles exhibit high stability in circulation, thereby harnessing the EPR effect. Guo et al. studied the potential of resveratrol (RES) and docetaxel (DTX) combination using methoxylpoly(ethylene glycol)-poly (d, L-lactide) (mPEG-PDLA) copolymer micelles in MCF-7 cells. The IC50 of RES and DTX in MCF-7 cells were found to be 23.0 μg/mL and 10.4 μg/mL, respectively, whereas a lower IC50 was obtained for the combination of resveratrol and docetaxel, which was 4.8 μg/mL. RES and DTX-loaded mPEG-PDLA micelles showed prolonged release profiles, as well as enhanced cytotoxicity against MCF-7 cells. The dual drug-loaded mPEG-PDLA micelles exhibited the highest anticancer activity against MCF-7 cells compared to other treatment groups of micelles containing either the same concentration of docetaxel or resveratrol [98]. Lapatinib (LP) is a drug that displays specificity for HER2-positive breast cancer cells. Moreover, LP is a dual tyrosine kinase inhibitor that interferes with the EGFr and HER2 pathways; nevertheless, its clinical use is limited owing to its high plasma protein binding and poor aqueous solubility. Therefore, a study by Wei Y et al. evaluated the effect of a novel polyethylene glycol and polylactic acid PEG–PLA micellar formulation of LP and paclitaxel (PPM-LP) on SKBr-3 (HER-2-positive) and MDA-MB-231 (HER-2-negative) cells. The findings show significantly increased cytotoxicity of PPM-LP against SKBr-3 cells compared to PPM–Paclitaxel, while there was no significant difference against MDA-MB-231 cells [57].

#### 4.2.3. Dendrimers

Dendrimers comprise compact and well-ordered nano-sized polymeric structures based on repetitively branched molecules or dendrons with a spherical, symmetric conformation around the particle core. Dendrimers have a well-defined size from 1–15 nm depending on the number of dendrons incorporated, and each dendron branch can incorporate active functional groups. The drug can either be encapsulated within the cavities of the dendritic polymer or be attached to functional groups via biodegradable linkers on the surface of the dendrimer. The drug can be released from the dendrimer whilst in circulation or internalised following its endocytic uptake. The well-defined, uniform, monodisperse, and highly branched symmetrical structure of dendrimers in contrast to conventional polymeric nanocarriers makes them attractive candidates for the targeted delivery of therapeutic and diagnostic agents [86]. For example, a study was carried out by Xue-Ling Guo et al. in which hyaluronic acid-modified polyamidoamine dendrimers were prepared for the systemic co-delivery of cisplatin and doxorubicin. Cell viability studies showed that the dendrimer elicited a higher anticancer effect on MCF-7 and MDA-MB-231 cells compared to the free drugs. Intravenous administration of the dendrimer to MDA-MB-231 tumour-bearing mice enabled selective accumulation of the drug at the tumour site, thereby significantly preventing tumour growth without appreciable toxicity [99].

Another study carried out by Mei, M et al. used a combination of chemotherapy and gene therapy. 5-fluorouracil (5-FU) was conjugated to polyamidoamine dendrimers by direct encapsulation combined with anti-sense micro-RNA 21 (as-miR-21), which can selectively knock down the expression of miR-21, a tumour oncogene overexpressed in primary breast cancer cells. The codelivery of as-miR-21 significantly enhanced the cytotoxicity and chemosensitivity of 5-FU, markedly increased the apoptotic percentage of the MCF-7 cells and reduced the migration ability of tumour cells [100]. Meng Wang et al. designed pluronic F68 (PF68)-conjugated polyamidoamine (PAMAM) dendrimer conjugates loaded with doxorubicin (DOX), aiming to overcome multidrug resistance in breast cancer. The antitumour activity of the DOX-loaded conjugates was evaluated against MCF-7/ADR cells, spheroids and tumours. The results revealed a significant increase in the antitumour activity of the DOX-loaded dendrimer conjugates both in vitro and in vivo. After cell entry via caveolae-mediated endocytosis, the DOX-loaded dendrimer conjugates escaped from the endosome/lysosome, thereby enabling nuclear uptake of doxorubicin. A significant increase in apoptosis regulated by mitochondrial function and gene expression was attained [62].

### 4.3. Lipid–Polymer Hybrid Nanoparticles

In recent years, advances have been made in the design of ‘hybrid’ nanocarriers that incorporate both polymeric and liposomal structures [101], variously known as polymeric lipid nanoparticles (PLNs) or lipid–polymer nanoparticles, with diameters of 100 nm or lower. In these hybrid structures, the polymer regulates drug release, whereas the lipid enhances the permeation of drugs across the cell membrane. Thus, polymeric lipid hybrid nanoparticles possess the dual advantages of the biomimetic characteristics of liposomes and the strength of biodegradable polymers, and are capable of improving the biocompatibility and physical stability of drugs, and boosting their potential for use in robust drug delivery. PLNs exhibit controlled drug release and high encapsulation for therapeutics with different properties, and can be targeted. There are various designs of PLNs ranging from a core–shell structure where a polymeric core is encapsulated by a lipid bilayer, or the polymer is instead attached to the bilayer surface, to a homogenous structure where the lipid phase contains a dispersed polymer drug complex.

Polymeric lipid hybrid nanoparticles hold many promising applications including cancer diagnostic imaging treatment [101]. PLNs were utilized for the codelivery of doxorubicin and mitomycin as a potential treatment of breast cancer by Zhang RX et al. [102]. The formulation was tested in a murine EMT6/WT breast cancer model. The results showed quickened and extended tumour uptake from fluorescence imaging of both agents over 24 h, and higher apoptosis and reduced organ toxicity were achieved with the PLN compared to free drugs [102]. In 2018, Monirinasab et al. synthesized PLA-PEG-PLA/cationic lipid hybrid nanoparticles for the delivery of insulin-like growth factor type I (IGF-1R) siRNA and examined their efficacy on MCF-7 cells. The results revealed high siRNA encapsulation efficiency (80%) and significantly higher cellular uptake of NPs/siRNA between 2–4 hcompared to the free siRNA and the control. Furthermore, treated cells exhibited significant downregulation and reduced expression of the IGF-1R gene (70%) in comparison to control cells [103]. In another study, aiming to achieve a synergistic effect through sequential and site-specific delivery, Jiang et al. fabricated a nanodepot, encapsulating doxorubicin in the interior, with the outer shell consisting of cross-linked hyaluronic acid entrapping a TNFα-related apoptosis-inducing ligand. Substantial inhibition of tumour growth was demonstrated in the MDA-MB-231 murine xenograft model [104]. The tumour-targeting potential of fructose-tethered PLNs co-loaded with methotrexate and beta carotene for the treatment of breast cancer was demonstrated by Jain et al. both in vitro and in vivo [70].

### 4.4. Biomimetic Nanoparticles in Breast Cancer

Conventional nanocarriers are susceptible to recognition by the immune system, which leads to a short circulation half-life and uptake by the reticuloendothelial system (RES). Surface modification of the nanoparticles by polyethylene glycol (PEG) can inhibit this process, but repeated use of PEG may also stimulate the production of its specific antibody and accelerate the clearance. Accordingly, this has stimulated research into biomimetic nanoparticles that recapitulate properties of natural materials, with the aim of countering immunogenicity and improving tissue uptake. Bioengineered components employed in cell-based drug delivery systems can include either whole cells, cell membranes or extracellular vesicles (exosomes) [105]. In the previous sections, we have described organic nanocarriers that are composed of substances that are chemically synthesized, and in the following section, we describe the various types of cell-based nanocarriers. In Table 3, we have listed examples together with their experimental application to breast cancer. In Table 4, we have tabulated the respective properties of conventional organic and cell-based nanocarriers, including certain advantages and disadvantages.

#### 4.4.1. Cell-Derived Nanoparticles

Cells can be utilized for drug delivery using several approaches, for example through the cell membrane coating of nanoparticles or codelivery of nanoparticles on cells (cellular trojan horses). Cell membrane coating technology comprises nanoparticles covered in a layer of cell membrane of various cell types such red blood cells, platelets, lymphocytes, cancer cells, stem cells and others. Owing to the presence of different proteins and carbohydrates on the membrane of various cell types, cells perform variable specific functions in the body and, therefore, the nanocarriers developed from cell-derived membranes that encapsulate drugs may incorporate some of the properties of the original cells. For example, red blood cells can escape the immune system due to the expression of CD47 on their cell membrane but have no targeting capability, while white blood cell membranes exhibit the characteristic of tumour homing [106]. Cell membrane-coated NPs (CMCNPs) are, therefore, increasingly being studied owing to their mimicry of cell surface properties and functions, in order to minimise immune responses compared to synthetic nanoparticles.

**Table 3:** Various biomimetic nanoparticles investigated in breast cancer.

Name of Nanoparticle	Composition/Coating of Nanodelivery System	Size	Drug/Biomolecule	Cell Line/Animal Model	Targeting	Outcome	Year	Reference
Albumin nanoparticles	Human Serum Albumin (HAS)	NA	Methotrexate (MTX) and transforming growth factor-β1 antibody (TGF-β1)	MDA-MB-231 cell line	Active targeting by folate	Increased cellular uptake of folate-HSA-MTX and TGF antibody on nanoparticles scavenged extracellular TGFβ1 of cancer cells, reducing cell migration.	2019	[107]
Albumin nanoparticles	Human Serum Albumin	246.5 nm	Curcumin (Cur)	MDA-MB-231, SK-BR-3, and MCF-7 cell lines	Active targeting by programmed death ligand 1 (PDL1) binding peptide for PDL1-overexpressing breast cancer cells	Peptide conjugation enhanced cellular uptake and cytotoxicity of HSA/Curcumin-loaded nanoparticles in PDL-1-overexpressing breast cancer cells.	2020	[108]
Albumin nanoparticles	Human Serum Albumin	175 nm	Doxorubicin and MDR1 siRNA	MCF-7 and MCF-7/ADR cell lines. MCF7/ADR tumour-bearing mice model	Active targeting by cetuximab for epidermal growth factor receptor	Cetuximab-targeted doxorubicin/MDR1 siRNA-loaded nanoparticles inhibited MCF-7/ADR cells’ proliferation through promotion of apoptosis and superior tumour inhibition vs. doxorubicin alone.	2021	[109]
Reconstituted high-density lipoprotein (rHDL) nanoparticles	Lecithin, cholesterol, cholesteryl oleate, triglycerides	101.3 nm	Paclitaxel (PTX) and HZ08	MCF-7, MCF7/PTX resistant to paclitaxel, MCF 10A and MCF or MCF7/PTX tumour-bearing model in mice	Active targeting of cells overexpressing scavenger receptor class B type I (SR-BI)	Paclitaxel-HZ08-rHDL nanoparticles showed significant enhancement of anticancer efficacy in vitro demonstrated by higher cytotoxicity and induction of cell apoptosis against both paclitaxel-sensitive and -resistant cell lines; stronger antitumour activity using nanoparticles vs. equivalent dose of paclitaxel.	2016	[110]
Reconstituted high-density lipoprotein (rHDL) nanoparticles	(2,3-Dioleoyloxy-propyl)trimethylammo-nium chloride, phospholipids and cholesterol	146–176 nm	Paclitaxel and siRNA VEGF (siVEGF)	MCF-7 cancer cell line/MCF-7 tumour-bearing mice model	Active targeting of cells overexpressing scavenger receptor class B type I (SR-BI)	In vitro results showed that rHDL/siVEGF-PTX caused a 14.96-fold increase in cytotoxicity compared to Taxol. In vivo studies demonstrated enhanced tumour growth inhibition.	2017	[111]
Reconstituted high-density lipoprotein (rHDL) nanoparticles	Dimyristoylphosphatidylcholine, Cholesterol oleate and ApoA-1 peptide	16.2 nm	Lenvatinib and vadimezan	4T1 cancer cell line/4T1 tumour-bearing mice	Active targeting of scavenger receptor class B type I (SR-BI) overexpressing 4T1 cells	In vivo results showed that LV-sHDL inhibited growth of 4T1 tumours, reduced lung metastasis and prolonged survival of animals.	2022	[112]
Human ferritin (HFn) nanocages	Heavy chain of human ferritin	14.3 nm	Curcumin	MDA-MB-468 and MDA-MB231 cell lines	Active targeting of Transferrin receptor 1 (TfR1)	More effective compared to free drug by abrogating the activity of multidrug resistance transporters.	2017	[113]
Human ferritin (HFn) nanocages	Heavy chain of human ferritin	15 nm	Paclitaxel	MDA-MB-231 cell line/MDAMB-231 tumour model in mice	Active targeting of Transferrin receptor 1 (TfR1)	HFtn-PTX nanoparticles showed significant cytotoxicity in vitro compared to free drug and had higher in vivo anticancer efficacy and lower systemic toxicity.	2019	[114]
Peptide-based nanoparticles	(C16-K(TPE)-GGGH-GFLGKPEG8, denoted as CTGP)	NA	Doxorubicin (DOX)	Cathepsin B-overexpressed MCF-7S and MCF-7R cell lines/MCF-7R tumour-bearing nude mice	Active targeting by cathepsin B-responsive peptide sequence (GlyPhe-Leu-Gly)	Efficient drug retention (46-fold of doxorubicin) and exceptional anti-MDR effect (50-fold of doxorubicin) in comparison to free drug as shown in in vitro and in vivo experiments.	2018	[115]
Peptide-based nanoparticles	Peptide amphiphile (PA) incorporating a TRAIL-mimetic peptide sequence	NA	Paclitaxel	MDA-MB-231 cells/tumour model in mice	Active targeting by binding of TRAIL-mimetic peptide sequence to death receptor 5 (DR5) overexpressed on cancer cells	High binding affinity to DR5-overexpressing cancer cells. When combined with paclitaxel, DR5-targeting nanoparticles showed potent antitumour activity in mice model.	2019	[116]
Nucleic acid-based nanoparticles	Staple DNA strands	NA	Doxorubicin and shPgp silencing P-glycoprotein and shSur silencing survivin	DOX-resistant human MCF7(MCF-7R) cell line/MCF-7R tumour-bearing mice	Active targeting by MUC1 aptamer	Results showed augmented synergistic antitumour effect against multidrug-resistant tumours both in vitro and in vivo.	2018	[117]
Nucleic acid-based nanoparticles	Branched DNA	100–140 nm	sgRNA/Cas9/antisense complex targeting tumour-associated gene polo-like kinase 1 (PLK1)	MCF-7 cancer cell line/MCF tumour xenograft model	Active targeting by adamantine-conjugated aptamer	Data revealed efficient tumour growth inhibition with undetected systemic toxicity.	2019	[118]
Nucleic acid-based nanoparticles	Staple DNA strands	NA	Doxorubicin	MDA-MB-468 and MDA-MB231 cell lines	Active targeting by folate	Higher uptake for targeted nanoparticles compared to nontargeted nanoparticles and the doxorubicin dose required to kill cancer cells was ∼31-fold lower for folate-functionalized nanoparticles.	2021	[119]
Nucleic acid-based nanoparticles	RNA oligonucleotides	NA	Anti-miR-21	MDA-MB-231 and MCF7/ADR cell lines	Active targeting by aptamer for epidermal growth factor receptor (EGFR)	RNA nanoparticles decreased cell viability and increased the sensitivity of breast cancer cells to doxorubicin in vitro. miR-21 inhibition by RNA nanoparticles caused the suppression of TNBC cell invasion, migration and colony formation.	2021	[120]
**Cell-derived nanoparticles: (a) Cell membrane-coated nanoparticles** 1. Cancer cell membrane (4T1 breast cancer cell membrane)	Polymeric nanoparticles Poly(caprolactone) (PCL) and pluronic copolymer F68)	175 nm	Paclitaxel	4T1 cells/4T1 tumour-bearing mice.	Active targeting binding of overexpressed VCAM1 adhesion molecule on cancer cells to monocytes expressing cell adhesion molecules such as α4β1 integrin	Higher cellular uptake of the cell membrane-coated nanoparticles and 36-fold greater cytotoxic efficacy compared to other groups. In the in vivo studies, cell membrane-coated nanoparticles exhibited the highest antitumour growth efficacy compared to the other treated groups.	2016	[121]
2. Cancer cell membrane (MCF7 cell membrane)	PLGA nanoparticles	202 nm	Curcumin and chlorin e6 (Ce6)	MCF-7 cell line/MCF-7 tumour-bearing mice model	Active targeting	Results showed significant cytotoxicity on MCF-7 cells of Cur/Ce6-cell membrane-coated NPs. In vivo data demonstrated prolonged circulation time, specific tumour accumulation and enhanced tumour growth inhibition compared to uncoated group.	2021	[122]
3. Red blood cell (RBC) membrane	Polymeric nanoparticles	148 nm	Paclitaxel	4T1 cell line/4T1 tumour-bearing murine model	Active targeting by binding of iRGD to cells overexpressing αvβ3 integrin and neuropilin-1	RBC-coated nanoparticles had a 5.8-fold higher elimination half time than that of the parental nanoparticles; nanoparticles significantly inhibited tumour growth and suppressed lung metastasis more efficiently than paclitaxel-loaded polymer nanoparticles alone or iRGD functionalized polymer nanoparticles.	2016	[123]
4. Red blood cell (RBC) membrane	PLGA nanoparticles	159 nm	Doxorubicin	MCF-7 epithelial cell adhesion molecule-positive (EpCAM+) cancer cell 2D and 3D spheroids	Active targeting by antiEpCAM antibodies	Results showed improved cytotoxic effect of targeted RBC NPs compared to nontargeted RBC NPs and free drug in both 2D and 3D in vitro breast cancer models.	2022	[124]
5. Macrophage cell membrane	Polymeric nanoparticles	NA	Paclitaxel	MDA-MB-231 cell line/tumour-bearing mice	Active targeting by insulin-like growth factor 1 receptor (IGF1R) peptide	In vitro and in vivo studies demonstrated that macrophage membrane-coated NPs resulted in the most extensive cell apoptosis among treated groups.	2018	[125]
6. Platelet membrane (PM)	PLGA nanoparticles	121 nm	Tumour necrosis factor (TNF)-related apoptosis-inducing ligand (TRAIL) and doxorubicin	MDA-MB-231 cell line/tumour-bearing mice	Active targeting by binding of P-selectin on platelet membrane and overexpressed CD44 receptors on cancer cells	Enhanced cytotoxicity compared with other treated groups at all studied TRAIL and doxorubicin concentrations, selective tumour targeting and higher tumour growth inhibition compared to doxorubicin.	2015	[126]
7. Platelet membrane (PM)	PLGA nanoparticles	293–300 nm	Doxorubicin and IR780 iodide	4T1 cell line/4T1 tumour model in mice	Active targeting by binding of P-selectin on platelet membrane and overexpressed CD44 receptors on cancer cells	Platelet membrane-coated NPs enhanced cancer cell killing in vitro and in vivo studies, showed accumulation at tumour site and enhanced tumour cell death upon NIR irradiation.	2020	[127]
**Cell-derived nanoparticles (b) Extracellular vesicles (Exosomes)**	Human bone marrow-derived mesenchymal stem cell (MSC) exosomes	128 nm	Paclitaxel	MDA-MB-231, MCF-7 cell lines/MDA-MB-231 tumour model in mice	Passive targeting	Reduced the viability of MDAMB-231 cells in vitro; significant tumour growth inhibition compared to control and/or MSC-EMs in vivo.	2018	[128]
Extracellular vesicles (Exosomes)	Cancer-derived exosome, HER2-positive SKBR-3 and EFM-192A cells and HER2-negative MCF-7 cells	30–300 nm	Trastuzumab emtansine (T-DM1)	HER2-positive SKBR-3 and HER2-negative MCF-7 cell lines	Active targeting by binding of trastuzumab to HER-2-positive breast cancer cells	T-DM1 enhanced binding to HER2-positive cancer cell-derived exosomes but not to exosomes derived from HER2-negative MCF-7 cells. Treatment of SKBR-3 and EFM-192A cells with T-DM1 containing exosomes caused tumour growth inhibition and activation of caspases 3 and/or 7	2018	[129]
Extracellular vesicles (Exosomes)	Monocyte-derived macrophages (THp1) exosomes	179 nm	Doxorubicin and miR159	MDA-MB-231); xenograft-breast tumour model in mice	Active targeting by binding of disintegrin and metalloproteinase 15 (A15) expressed on exosomal membranes to integrin αvβ3 on cancer cells	Co-loading of doxorubicin and miR159 to A15-Exo generated synergistic therapeutic effects in vitro. Delivery of miR159 and doxorubicin effectively silenced the TCF-7 gene, resulting in better anticancer effects without noticeable side effects.	2019	[130]
Extracellular vesicles (Exosomes)	Adipose-derived mesenchymal stem cells	40–100 nm	miR-381	MDA-MB-231 cell line	Passive targeting	miR-381-loaded exosomes significantly downregulated expression of epithelial to mesenchymal transition (EMT)-related genes and proteins, which led to inhibition of proliferation and migration of MDA-MB-231 cells in vitro.	2021	[131]
Extracellular vesicles (Exosomes)	Cancer-derived exosomes (SKBR-3 and MCF-7 cells)	65 nm	Recombinant P53 protein	SKBR-3, MCF-7 cell lines/4T1 tumour model in mice	Active targeting of mitochondria by triphenylphosphonium (TPP)	Findings revealed successful targeting of PP/P53 to mitochondria of breast cancer cells. In vivo results showed good tumour accumulation and destruction without apparent systemic toxicity.	2022	[132]

**Table 4:** Summary of properties of different types of organic nanoparticles.

Nanoparticle	Properties
Liposomes	Biocompatible
	Biodegradable and can be stored in lyophilised form or as an aqueous suspension
	Versatile carrier of neutral, hydrophobic and hydrophilic agents
	Improved pharmacokinetics using PEGylation renders liposomes suitable for
	passive targeting; active targeting via surface ligands
Polymeric nanoparticles/micelles	Biocompatible and generally biodegradable
	Nanocarriers for hydrophobic agents, but less suitable for hydrophilic agents
	Can offer high drug loading capacity (copolymer micelles)
Solid lipid nanoparticles	Biocompatible
	Biodegradable but low-temperature storage required with current formulations
	Incorporation of agent within solid lipid matrix affords protection against
	degradation, e.g., hydrolysis
Dendrimers	Branched, well-defined polymeric structure that can accommodate hydrophobic
	agents within or surface-conjugated hydrophilic agents
	More complicated structurally than other polymeric nanocarriers; therefore,
	synthesis is more expensive, and only limited or no biodegradability
	Suitable for passive (large dendrimers) and active targeting
Biomimetic nanoparticles (e.g., exosomes)	Biodegradable and versatile nanocarriers
	Very low immunogenicity with ability to traverse physiological barriers
	Scaled-up production of biomimetic exosomes is difficult and long-term storage
	requires low temperatures

Applications of cell-derived nanoparticles in experimental breast cancer treatment are summarised in a separate section of Table 3. Cell- and cell membrane-based drug carriers offer certain advantages compared to other drug delivery systems, which has stimulated their use in cancer, such as: (a) evasion of the immune system and longer circulation time; (b) tumour targeting and EPR effect enhancement capabilities provided by the cell membrane proteins; (c) ability to produce cytotoxic immunomodulatory effects. A further potential benefit of utilizing CMCNPs is the circumvention of drug resistance caused by tumour heterogeneity [133]. A tumour-homing cell membrane can help nanoparticles better overcome the various obstacles to tumour uptake including uptake by tumour-associated macrophages. In a study by Tian et al., PLGA nanoparticles loaded with paclitaxel were coated with mesenchymal stem cell membranes were tested using a murine orthotopic breast cancer model. Prolonged circulation was observed, together with controlled drug release, eliciting effective tumour uptake [134]. Kang et al. used neutrophil membrane-coated polymeric nanoparticles loaded with carfilzomib, a proteasome inhibitor, for treatment of a lung metastatic 4T1 model, and elicited targeted delivery to the metastases [135]. Platelet-coated PLGA nanoparticles overexpressing P-selectin were also used to deliver tumour-specific apoptosis-inducing ligand cytokine (TRAIL) and doxorubicin to MDA-MB-231 tumour cells in a murine animal model due to the specific binding of P-selectin to the CD44 receptors expressed on the tumour cells. As a result of the enhanced targetability between the platelet membrane and cancer cells, the therapeutic efficacy of TRAIL and doxorubicin was improved, resulting in increased apoptosis and tumour growth inhibition [126]. Pan et al. manufactured platelet-coated liposomes expressing two peptides, GPIIb-IIIa-like integrins and P-selectins, on their surface, which were loaded with doxorubicin and elicited enhanced targeting of metastases in vivo [136].

#### 4.4.2. Extracellular Vesicles (EVs)

Extracellular vesicles or exosomes are identified as membrane-bound biological nanoparticles that can be involved in intercellular signalling either in physiological or pathological conditions. EVs are generated by virtually all cell types, and are composed of various biomolecules according to their origin, e.g., membrane proteins, lipid bilayers, metabolites and nucleic acids. Membrane proteins play a role in targeting EVs to particular sites, whereas lipid bilayers aid in protecting bioactive contents from the body fluid environment and degradation by extracellular proteases and nucleases. Extracellular vesicles can dissociate from the source cells and spread through the vascular system, targeting specific cells to deliver their contents. Extracellular vesicles have attracted interest for the targeted delivery of imaging therapeutic agents due to their cell-derived origin [137]. Extracellular vesicles have several advantages compared with conventional nanocarriers. Firstly, owing to their natural phospholipid bilayer structure, they can extend the circulation time of incorporated drugs and confer protection against degradation whilst in circulation. Secondly, compared with synthetic drug carriers, extracellular vesicles are found to exhibit lower immunogenicity, and may also facilitate tumour treatment by regulating immunity [138]. However, the difficulty of large-scale reproducible preparation of EVs remains to be resolved before these advances can be fully exploited.

Tian et al. studied the delivery of doxorubicin to tumour tissue in murine MDA-MB-231 and MCF-7 models using engineered exosomes. In this study, immature dendritic cells (imDCs) were genetically modified to express lysosome-associated membrane glycoprotein 2b (Lamp2b), a well-characterized exosomal membrane protein, linked to the iRGD peptide that targets αv integrin expressed on tumour cells. The efficacy and improved therapeutic index of the iRGD-exosomes loaded with doxorubicin was demonstrated in vitro and in vivo via the specific targeting of αv integrin-expressing cells (MDA-MB-231 cells), resulting in tumour growth inhibition without noticeable toxicity [139]. Another study carried out by Ohno et al. explored the use of modified exosomes with the GE11 peptide or EGF on their surfaces to deliver miRNA to EGFR-expressing breast cancer tissues. GE11-targeted exosomes exhibited three-fold higher uptake compared to control exosomes [140].

#### 4.4.3. Nucleic-Acid-Based Nanostructures

Nucleic acids (DNA and RNA) are natural building blocks that enable a bottom-up self-assembly approach stemming from precise base pairing that can be exploited for the manufacture of well-defined nanostructures. The introduction of biological tags and stimuli-responsive moieties into DNA or RNA structures has enabled the exploitation of their properties beyond their natural roles and made it possible to design nanostructures for many applications, including cancer targeting and therapy [141]. A key advantage is that functional molecules can be incorporated at specific sites in the nucleic acid structure to optimise the formulation for the desired application. Nucleic-acid-based nanostructures can utilize the targeting abilities of DNA and RNA to interact with cellular components. For instance, aptamers, which are single-strand DNA or RNA molecules (20–100 nucleotides) exhibit high targeting affinity and specificity, and the integration of aptamers with nucleic acid nanostructures can be used to target therapeutics to cancer cells or to tumour biomarkers. Moreover, nucleic acid nanostructures can be designed with particular geometric shapes (for example, triangular DNA origami), which also incorporate environmentally responsive structural properties [141]. Zhao et al. investigated DNA nanostructures for the optimal delivery of doxorubicin to MDA-MB-231, MDA-MB-468, and MCF-7 cells. These nanostructures could incorporate variable degrees of twist, enabling the tuning of encapsulation efficiency and drug release [142]. In a similar study, Zhang et al. confirmed the previous finding reported by Zhao et al. (2012) but in an in vivo experiment using murine MDA-MB-231-GFP orthotopic breast tumours. In this study, triangular DNA origami vehicles were used to deliver doxorubicin (DOX). In comparison to free DOX, the complex elicited more effective and selective tumour damage without systemic side effects [143]. In a different study by Shuai et al., a novel DNA origami nanostructure delivery platform was designed to enable the delivery of cytotoxic protein RNase A. These nanocarriers were decorated with 32 anti-MUC1 DNA aptamer strands, which target mucin 1 (MUC1), a glycoprotein overexpressed on the surface of MCF-7 cells. The study showed that the loaded DNA origami nanostructures were the most efficient in killing MCF-7 cells among all the treated groups [144]. In another study, Zhang et al. synthesized a DNA–affibody nanoparticle for targeting the HER2 receptor. The nanoparticle had a smaller size (95 kDa) compared to the HER2 binding monoclonal antibody, trastuzumab (150 kDa), and exhibited two-fold higher activity against BT474 cells compared to the antibody. The DNA in this nanostructure enabled both the attachment of two affibody molecules and binding for drug delivery. Each DNA–affibody nanoparticle could bind approximately 53 molecules of doxorubicin to provide an effective targeted delivery system [145].

As with DNA, RNA can be used for the fabrication of nanoparticles, which can be functionalized with targeting moieties, imaging and therapeutic agents, which, in some cases, can also be composed of RNA nucleotides [146]. In cancer therapy, microRNA (miRNA) plays an important role in regulating the gene expression and survival of cancer cells, which has stimulated interest in their use as therapeutic targets. Shu et al. utilized RNA nanotechnology to deliver anti-miRNA with the aim of inhibiting the proliferation of triple-negative breast cancer cells in mice. Multifunctional RNA nanoparticles were prepared, consisting of an EGFr-targeting ligand based on RNA aptamers, anti-miRNA as the therapeutic agent and a fluorescent dye for imaging. In vivo studies confirmed selective tumour uptake [147]. In another study using a triple-negative breast cancer model, Guo et al. prepared RNA nanoparticles loaded with paclitaxel and targeted with an anti-EGFR aptamer, which effectively inhibited tumour growth [148].

#### 4.4.4. Protein-Based Biomimetic Nanocarriers

As a result of their versatility and biocompatibility, proteins have gained considerable attention as templates for drug delivery nanosystems and have been used successfully for the delivery of anticancer drugs and other agents [149]. The application of different types of protein-based nanocarriers for experimental breast cancer treatment are summarised below.

##### Serum Albumin Fabricated Nanoparticles

Albumin is a highly soluble, strong protein that remains stable at pH 4–9. Moreover, the selective uptake of albumin in tumour and inflamed tissues and its biocompatibility, low toxicity, biodegradability and low immunogenicity make it suitable for systemic in vivo applications. Additionally, albumin contains many functional groups that can be suitable for the binding of molecules, making it an attractive substrate for nanoparticle preparation. Moreover, tumour metastases may express albumin-binding proteins, thereby improving the uptake of the albumin-based nanoparticle [150]. Taking into account albumin’s abundance, low cost, ease of purification and unique binding properties, albumin-based nanoparticles are considered promising candidates for drug delivery. Bovine serum albumin (BSA) is a commercially available protein that has been widely used in the formulation and preparation of nanoparticles. However, BSA presents the risk of immunogenicity in clinical use; therefore, studies have focused on human serum albumin (HSA) [105]. For example, Wan et al. developed lapatinib-loaded HSA NPs, which inhibited the migration and invasion ability of 4T1 cells more effectively versus the free drug in vitro [151]. In another study using the same cell line model, Yi et al. investigated HSA NPs coloaded with pirarubicin (THP) and paclitaxel (PTX) (Co-AN). The prepared nanoparticles showed high loading efficiency and controlled release, and higher tumour drug accumulation and antitumour efficacy with reduced uptake in normal tissue were observed for Co-AN [152]. Saleh et al. investigated targeted delivery to HER2-overexpressing breast cancer cells by synthesizing HER2 aptamer-functionalized curcumin-loaded nanoparticles. Cytotoxicity studies elicited no significant difference between the cytotoxic effect of free curcumin and non-targeted HSA/curcumin nanoparticles in both HER2-positive and HER2-negative cell lines, whereas NPs resulted in significantly higher cytotoxicity in SK-BR3 cells [153]. A study reported the design of human serum albumin nanoparticles conjugated with transforming growth factor (TGF)-β1 antibody, methotrexate (MTX) and cancer-targeting folic acid (FOL). The results revealed higher cellular uptake and cytotoxicity of FOL-HSA-MTX by MDA-MB-231 cells overexpressing folate receptors [107].

##### Ferritin-Based Nanocarriers

Ferritin is a ubiquitous intracellular protein for storing iron. A key property of ferritin is that it can self-assemble into a cage-like nanostructure with an external diameter of 12–13 nm comprising 12 or 24 subunits, and this architectural formation of ferritin has attracted much interest in its use as a nanomaterial. Additionally, ferritin is differentially overexpressed in several types of malignant tissue including breast cancer, and it was found that L-ferritin was approximately six-fold higher in breast tumours than benign breast tissue [105]. H-chain ferritin (HFtn) is specifically recognized by the transferrin receptor-1 (TfR1), which is overexpressed in various human cancer types, including triple-negative breast cancer. Transferrin receptor-1 promotes the cellular internalization of these nanoparticles. Paclitaxel was encapsulated into the cavity of HFtn nanocages, and the cellular uptake and antitumour effect were evaluated using flow cytometry, confocal laser scanning microscopy and in vivo imaging of MDA-MB-231 tumours. The findings showed that HFtn-paclitaxel nanoparticles elicited greater therapeutic efficacy and lower systemic toxicity in vivo [114]. Another study reported the fabrication of paclitaxel (PTX) loaded with extracellular-signal-regulated kinase (ERK) peptide inhibitor in apoferritin nanocages. The ERK peptide inhibitor can interrupt the interaction of MEK with ERK in the mitogen-activated protein kinase/ERK pathway, which is involved in the proliferation of cancer cells. By combining the advantage of targeted delivery provided by ferritin and the inhibitory effect of the ERK peptide inhibitor, the newly manufactured ferritin nanocarrier HERK could still be taken up by cancer cells, displaying greater cytotoxicity in MDA-MB-231 cells than the parent ferritin. A remarkable inhibitory effect on MDA-MB-231 tumour spheroids was demonstrated as well [154]. A novel nanoformulation consisting of H-Ferritin nanoparticle loaded with Olaparib (HOla) was developed, examined and compared to free Olaparib for in vitro efficacy on three different triple-negative breast cancer cell lines. Olaparib acts by inhibiting Poly (ADP-ribose) polymerase (PARP-1), an enzyme involved in DNA repair. The aim of this study was to optimize the delivery of Olaparib through the encapsulation of the drug into a nuclear-targeted HFn nanocarrier. Delivering the drug to cancer nuclei via HOla nanoparticles is expected to enhance the anticancer effect in both BRCA-mutated and sporadic triple-negative breast cancer. HOla exhibited a notable antitumour effect compared to Ola in all triple-negative cell lines, with maximal differences at 100 nM. HFn nanocages exploited the natural tumour targeting mediated by transferrin receptor-1 (TfR1). TfR1-overexpressing triple-negative cell lines were successfully targeted by H-Ferritin, and HOla induced significant cytotoxicity that was 1000-fold higher compared to free Olaparib [155].

##### Lipoprotein-Based Nanocarriers

High-density lipoproteins (HDLs) and low-density lipoproteins (LDLs) are examples of lipoproteins that form through the spontaneous synergistic self-assembly of lipids combined with a type of protein known as apolipoproteins. Considering that the size of the LDL and HDL (less than 30 nm) can promote the deep penetration of lipoproteins into tumours, biocompatible lipid complexes could also be ideal candidates for cancer diagnosis and therapy. HDL has gained attention as a nanodelivery system due to its biodegradability, low immunogenicity and natural targeting ability. The hydrophobic core and phospholipid corona of HDL facilitate the incorporation of lipophilic cargoes, imaging agents and therapeutic drugs. Moreover, HDL particles can evade the reticuloendothelial system and subsequently exhibit prolonged circulation times. In a study by Gong et al., a high-density lipoprotein (sHDL) nanoparticle loaded with docetaxel (DTX) was manufactured and tested against breast cancer. The data revealed that the synthesized sHDL nanoparticles displayed a high loading efficiency of docetaxel, slow drug release profile and excellent biocompatibility. Furthermore, DTX-sHDL nanoparticles improved the uptake of docetaxel, augmented the cytotoxic effect against MCF-7 cells and reduced the toxicity to normal cells. Finally, in vivo experiments in 4T1 tumour-bearing mice showed superior tumour growth inhibition by DTX-sHDL nanoparticles than that of the free docetaxel-treated group [156]. Another study reported the design of a peptide-functionalized dual-targeting delivery system encapsulating paclitaxel and GANT61 in tLyP-1 peptide-modified reconstituted high-density lipoprotein nanoparticles (tLyP-1-rHDL-PTX/GANT61 nanoparticles), which may show potential when used in combination with conventional chemotherapy for treating metastatic triple-negative breast cancer. GANT61 is an inhibitor of the sonic hedgehog (SHH) signalling pathway, which regulates several malignant phenotypes related to tumour metastasis, including triple-negative breast cancer. However, it exhibits poor bioavailability and off-target toxicity, which prompted investigating the use of a targeted high-density lipoprotein nanoparticle. This was achieved by conjugating a high-density lipoprotein nanoparticle to the apolipoprotein A-1 and tLyP-1 peptides that bind to the overexpressed scavenger receptor B type I and neuropilin-1 receptor. The prepared nanoparticles showed higher cellular uptake in both MDA-MB-231 and BT-549 tumour cells, and their 3D tumour spheroids. In vitro experiments revealed that GANT61 was capable of suppressing crucial tumour cell activities related to metastasis such as stemness, angiogenesis, migration and invasion. The codelivery of paclitaxel and GANT61 by nanoparticles elicited high tumour-specific accumulation and significant primary tumour growth inhibition, together with a reduction in lung metastasis without noticeable side effects using the spontaneous murine metastatic NCG breast model [157].

##### Peptide-Based Nanocarriers

Peptides are attractive substrates for biocompatible and degradable nanodelivery systems and other biomedical applications such as biosensors and tissue regeneration. The self-assembling properties of peptides have been exploited for the fabrication of nanostructures of different shapes, for example nanotubes, nanofibers and hydrogels [158]. Various types and structures of peptides have been used to self-assemble into nanostructures such as amphiphilic peptides, dipeptides, cyclic peptides and sheet peptides. Adjusting the basic units of peptides aids in synthesizing the desired molecular structures. Cyclic peptides composed of alternating D-type and L-type amino acids that can self-assemble into nanotubes have distinctive features compared to other types of peptide self-assembled nanostructures such as the ability to precisely control the diameters by tuning the peptide sequences and lengths. Modifying the peptide side chains could further tune the functions of the nanotubes [159]. For instance, cyclic peptide self-assembled nanotubes have been constructed using eight-residue cyclic peptides comprising Glu and Cys amino acids. The results suggested that PEG-modified doxorubicin-loaded nanotubes had a high drug encapsulation ratio. Furthermore, these nanotubes have demonstrated enhanced doxorubicin uptake in MCF-7/ADR cells and higher cytotoxicity in vitro compared to free doxorubicin. Additionally, PEG-modified doxorubicin-loaded nanotubes enhanced the inhibition of P-gp activity in MCF-7/ADR cells, which demonstrates their potential to overcome the multidrug resistance in tumour therapy [160]

### 4.5. Multifunctional Nanostructures and Tumour-Targeted Delivery

Nanostructures can be designed to be multifunctional so that they can incorporate therapeutic agents, targeting ligands, imaging agents, stimuli-responsive structures, and molecules that can control the drug release profile, all within the same nanosystem. Surface functionalisation by molecules that inhibit recognition by the immune system is another important feature, and in addition to the aforementioned approach of PEGylation, a number of other strategies have been investigated such as coating with peptides or polysaccharides [141]. In this section, the design of multifunctional nanoformulations is considered in more detail for their applications in tumour-targeted drug delivery and for stimuli-responsive therapy.

#### 4.5.1. Tumour-Targeted Delivery

The tumour microenvironment (TME) comprises cellular and non-cellular components that form the tumour’s niche. The non-cellular components include the extracellular matrix, growth factors, cytokines, chemokines, small RNAs, DNA and extracellular vesicles. Along with cancer cells, several other cell types are also present in the tumour tissue for example mesenchymal stromal cells, adipocytes, pericytes, cancer-associated fibroblasts, cancer stem cells and immune cells such as tumour-associated macrophages (TAM), T and B lymphocytes, dendritic cells (DC), neutrophils and natural killer cells (NK). The tumour microenvironment is characterized by the presence of hypoxia, acidosis and oxidative stress, and by interactions between these different cell types, which will cause modulation of the immune system, induction of angiogenesis and subsequent metastasis. Several barriers in the tumour microenvironment have been found to inhibit the diffusion of NPs through the tumour tissue, such as dense extracellular matrix, high interstitial fluid pressure and the heterogeneous permeability of blood vessels. Hence, to improve treatment efficacy, a number of new strategies that can deliver nanoparticles deep into tumours [161] have been developed. Moreover, the binding of drugs and nanoparticles to components within the tumour extracellular matrix offers an alternative means of targeting the cancer cells, as described below.

##### Targeting the Extracellular Matrix

Activated cancer-associated fibroblasts and mesenchymal stromal cells secrete extracellular matrix components, cytokines and chemokines that stimulate tumour cell proliferation and drug resistance. Stromal cell proteins such as fibroblast activation protein α and extracellular matrix components (particularly extracellular matrix enzymes) can, therefore, be utilized as targets for delivery or a method for selective drug release. In order to enhance the penetration and targeting of nanoparticles, their coupling with drugs that target stromal aberrations within the tumour is one such approach that has been proposed [161]. Ji, T et al. synthesized a new amphiphilic peptide (CAP) that can be cleaved by fibroblast activation protein-α (FAP-α), a protease particularly expressed on the surface of cancer-associated fibroblasts. In the proposed solution, CAPs self-assembled into fibre-like nanostructures, which were transformed into spheroidal nanoparticles encapsulating chemotherapeutics such as doxorubicin. The disassembly of these nanoparticles upon FAP-α cleavage led to the efficient release of the encapsulated drugs at tumour sites, and superior therapeutic efficacy for a variety of solid experimental tumours including xenograft MCF-7 breast tumour models [162]. Another study by Hao Zhou et al. exhibited the conjugation of recombinant human hyaluronidase PH20 (rHuPH20), which degrades hyaluronic acid, on the surfaces of PLGA-PEG nanoparticles. Surface modification with rHuPH20 resulted in a four-fold increase in the accumulation of PLGA-PEG nanoparticles in 4T1 syngeneic mouse breast tumours with a uniform tumour distribution. Encapsulating doxorubicin in the nanoparticles successfully inhibited breast tumour growth [163]. Another study by Li X et al. utilized sulphatide, which binds to the extracellular matrix glycoproteins overexpressed in breast tumours. In this study, sulphatide and lipid perfluorooctylbromide nanoparticles were synthesized with the aim of targeted breast cancer delivery vehicle for paclitaxel. Growth inhibition experiments in an EMT6 xenograft tumour model showed that these nanoparticles were significantly more potent vs. the free drug [164].

Fibronectin is a glycoprotein that is abundant in the extracellular matrix and upregulated in many tumours, which can be exploited for drug targeting using antibodies and peptides. CREKA (Cys-Arg-Glu-Lys-Ala) is an example of a targeting peptide that binds to the fibrin–fibronectin complex. PEGylated liposomes containing CREKA loaded with doxorubicin elicited higher retention in murine 4T1 breast tumours but negligible binding to fibrin–fibronectin complexes in normal tissue [165]. In the tumour microenvironment, cytokines such as tumour necrosis factor (TNF-a), interleukins (IL-1, IL6 and IL-10) and transforming growth factor b (TGF-b) contribute significantly to variable activities in tumourigenesis including cancer-associated chronic inflammation. Combinations of anti-inflammatory agents can improve the effectiveness of nanomedicines. Zuo et al. showed that the inhibition of TGF-b signalling pathway by the TGF-b type I receptor inhibitor LY364947 enhances the tumour penetration of the nanoparticles carrying siRNA targeting breast cancer stem cells. This combined treatment synergistically inhibited tumour growth and improved cancer stem cell clearance in breast cancer mouse xenografts [166].

##### Targeting Breast Cancer Cells

To enable tumour homing in breast cancer tumours, nanoparticles can be functionalized with general tumour-homing peptides or proteins specifically recognizing breast cancer epitopes. The tripeptide RGD is a common tumour-homing peptide found in proteins that mediate cell adhesion. This peptide is recognized and bound by integrins present in tumour cells with high affinity. It has gained much interest because it targets both breast tumour and tumour angiogenesis. Integrins are receptors abundantly expressed on cancer cells and associated blood vessels, mediating cell motility, proliferation and apoptosis. Breast cancer cells are found to express α6β4 and ανβ3 integrins, which have been associated with increased tumour size, grade and metastasis. Attaching RGD peptides to nanoparticles to target breast cancer cells for imaging and treatment purposes has been explored on multiple occasions [167]. Cyclic RGD octapeptides have been used to target rapamycin and doxorubicin-loaded liposomes to integrin α3 in MDA-MB-231 (TNBC) xenografts, demonstrating significantly higher tumour accumulation [168]. In another study, Ma et al. developed an integrin α3-targeted nanoformulation containing bortezomib, a known anticancer drug, to image and treat MDA-MB-231 cells [169]. Human epidermal growth factor receptor-2 (HER2) is expressed markedly (100–1000 times higher than normal breast cells) to preserve the aberrant growth of breast cancer cells. Studies reported that around 25–30% of breast cancer cases have higher expression of the HER2 gene that is associated with multidrug resistance and tumour recurrence. Accordingly, HER2 is considered a promising target for drug delivery to HER2-positive breast cancer. Among the monoclonal antibodies successfully targeting breast cancer and routinely used for treatment clinically are the anti-HER2 antibodies Trastuzumab and Pertuzumab. Unfortunately, resistance to antibodies such as trastuzumab has developed recently in some breast tumours. Therefore, the application of nanotechnology has been investigated to counteract this effect [167]. A study carried out by Niza et al. reported the fabrication of trastuzumab-targeted Dasatinib (DAS)-loaded nanoparticles. Dasatinib is a multikinase inhibitor that acts on many signalling kinases, and is active against human epidermal growth factor receptor 2 (HER2)-positive tumours. In vitro studies were carried out on HER2-positive BT474 and BT474-RH (Trastuzumab-resistant) cells and triple-negative MDA-MB231 cells. The trastuzumab-conjugated DAS-loaded nanocarriers induced cytotoxicity in HER2-overexpressing cell lines versus DAS, and in BT474-RH cells, trastuzumab-DAS-NPs exhibited more activity than each agent alone and control NPs [170]. Another study carried out by Zhang et al. prepared trastuzumab (Tmab)-coated lipid–polymer hybrid nanoparticles (PLNs) loaded with docetaxel. Tmab-docetaxel PLNs exhibited increased cytotoxicity in HER2-postive BT474 cells but not in HER2-negative MCF-7 cells [171].

Epidermal growth factor receptor (EGFR) is overexpressed in approximately 50% of triple-negative breast cancer cases. The antibody anti-EGFR Cetuximab successfully targets EGFR-overexpressing cancers, specifically in triple-negative breast cancer. For example, Roncato et al. conjugated Cetuximab to nanoparticles and nucleic acid assemblies carrying doxorubicin to selectively target triple-negative breast cancer MDA-MB-231 cells [172]. Another study investigated cetuximab-modified HAS nanoparticles co-loaded with doxorubicin and multidrug resistance 1 silencing RNA (siRNA) (C-H/D/M) for the treatment of drug-resistant breast tumours (MCF-7/ADR). The results showed that C-H/D/M significantly enhanced and maintained the sustained release and the uptake of doxorubicin. Additionally, MDR1 mRNA and P-gp expression levels were downregulated. The successful tumour-targeted delivery of DOX and siMDR1 by C-H/D/M was confirmed through confocal imaging together with tumour growth inhibition [109]. In order to improve drug efficacy by overcoming tumour cell receptor heterogenicity, Houdaihed et al. formulated a polymeric nanoparticle functionalized with Fab fragments targeting both HER2 and EGFR receptors for the delivery of paclitaxel (PTX) and Everolimus (EVER) in SK-BR-3 (HER2-positive/EGFR moderate) breast cancer cells and spheroids. The dual-targeting nanoparticles induced a spheroid size reduction approximately 84% greater than the untargeted nanoparticles [173].

##### Targeting Breast Cancer Subcellular Organelles

The fundamental difference between nanoformulations and free drugs is the controllable release of the cargoes in specific locations. Some drug formulations are not simply delivered to the cell interior, but require direct delivery to the subcellular site/organelle of action (e.g., nucleus, lysosome, mitochondria, endoplasmic reticulum, Golgi body). This direct delivery of therapeutic agents to their site of action has the potential to amplify the therapeutic efficacy of the agent, but the sole use of cell surface biomarkers is unlikely to be sufficient to deliver drugs to particular subcellular locations/organelles [174]. Consequently, recent research has focused on developing advanced nanocarriers that can also target the intracellular site of action following internalisation. This strategy of subcellular targeting and, in certain cases, drug release depends on the characteristics of the microenvironment in various organelles, such as the acidity of lysosomes, the weak basicity of the endoplasmic reticulum and the high expression of ROS and H_2_O_2_ in the mitochondria. Subcellular targeted nanoparticle–drug systems can be designed to have dual targeting (cell-type specificity and subcellular localization) capability combined with triggered release of the payload [174]. A recent study reported the development of a novel sequential targeted core–shell nanoparticles targeted via hyaluronic acid to the CD44 receptor (overexpressed in breast cancer cells) in tandem with subcellular mitochondrial drug delivery using the mitotropic triphenylphosphonium-conjugated doxorubicin, which were incorporated into chitosan-based nanoparticles [175].

Wang et al. described the formulation of a multifunctional micelle system (HEKM) composed of shape-altering micelles incorporating doxorubicin, a metalloproteinase (MMP-2) substrate, an optical imaging agent, a mitochondrial damage segment (KLA) and a peptide targeting the EGFR/HER2 complex that is overexpressed in certain breast tumours. Studies were carried out using SKBR-3 and MDA-MB-468 carcinomas that highly express EGFR-HER2. HEKMs initially self-assembled as nanorods in the circulation, which may aid the avoidance of uptake by the reticuloendothelial system. When HEKMs reached the tumour area, guided by binding to the EGFR-HER2 complex, the MMP-2 substrate was degraded (as confirmed through fluorescence energy transfer analysis), leading to the deformation of the nanorods into spheres, which aided deeper tumour penetration. The HEKM-mediated combination therapy elicited the highest tumour growth inhibition amongst the treated groups, consistent with improved targeting and tumour penetration [176]. Another study reported an interesting nuclear-targeting strategy to counter multidrug resistance using liposomes, where the aptamer AS1411 was co-encapsulated with doxorubicin. After internalization, the aptamer–doxorubicin complex was released from the liposomes and migrated to the nucleus through the aptamer–nucleolin interaction, which facilitated the evasion of doxorubicin efflux by P-gp pumps, resulting in improved therapeutic efficacy [177].

#### 4.5.2. Stimuli-Responsive Nanoparticles

Nanoparticles can be functionalized with stimuli-responsive materials, which confers new properties through altering their physicochemical behaviour in response to small external or internal stimuli and increasing the selectivity within the tumour tissue, and exhibits advantages compared to conventional systems, namely a reduction in the systemic toxic effects. These materials can be divided into intrinsic/chemical (pH, hypoxia, redox and enzyme) and extrinsic/physical (ultrasound, light, magnetic field, near infrared and electric). In this way, stimuli-responsive nanoparticles can achieve, for example, the release of drugs in a specific tissue in response to the applied or intrinsic stimulus [178].

##### pH-Responsive Nanoparticles

Nanocarriers that are responsive to changes in pH have been commonly used as an approach for drug delivery in cancer therapy by exploiting the observation that tumours possess a lower pH (5.4 to 6.8) than adjacent normal tissue. This characteristic of tumour tissue is attributed to the high rate of glycolysis in cancer cells and excessive production of acidic compounds such as lactate. For breast cancer therapy, a variety of “smart” pH-sensitive nanocarriers have been designed. For instance, a study by Zhou et al. reported the synthesis of a multifunctional polymeric nanocarrier incorporating a targeting ligand and pH-sensitive moiety to increase the specificity and efficacy of the drug delivery system. Trastuzumab was conjugated to the nanocarriers, and encapsulation of paclitaxel and doxorubicin was achieved. In vitro experiments against breast cancer cell lines showed significantly increased uptake for trastuzumab-conjugated nanoparticles compared to non-targeted nanoparticles, and they exhibited superior therapeutic efficacy in vivo [179]. Another study reported the design and preparation of polymeric nanoparticles containing polylysine and PEG for the synergistic treatment of breast cancer by encapsulating lapatinib and doxorubicin into pH-responsive/charge-switchable nanoparticles [180]. The nanoparticles were stable in the circulation, but their surface charge switched from anionic to cationic in the acidic tumour microenvironment, thereby enhancing the tumour localization and subsequent release of both drugs, which elicited complete tumour eradication.

##### Hypoxia-Responsive Nanoparticles

Hypoxia is commonly known to be associated with all types of solid tumours. It occurs due to overconsumption of oxygen by rapidly proliferating cells present at the periphery of the tumour, leaving only a small portion of oxygen remaining to diffuse further into the tumour centre. Hypoxia can also arise from immature and leaky tumour blood vessels and poor perfusion, and these hypoxic areas are much less susceptible to chemotherapy owing to the limited drug delivery via the microvasculature. Radiation therapy is less effective at treating hypoxic areas due to the lower generation efficiency of oxygen free radicals, which cause cytotoxic DNA damage. Nanotechnology research has, therefore, focused on exploiting this physiological condition to develop nanoparticles, which are activated only when they encounter hypoxic conditions whilst remaining inactive under normoxia [178]. Numerous hypoxia-responsive polymeric nanoparticles have been formulated for drug delivery through the incorporation of hypoxia-sensitive groups, for instance nitrobenzyl alcohols, nitroimidazoles and azo derivatives. These hypoxia-responsive moieties can generate hydrophilic functional groups in response to the hypoxic microenvironment, enhancing the hydrophilicity of the nanocarriers and resulting in efficient drug release under hypoxic conditions. For example, a study has reported a hypoxia-sensitive polymer, mPEGPLG-NC, that can self-assemble into micelles in aqueous media and doxorubicin with high efficiency but demonstrated drug release when exposed to hypoxia. Studies in 4T1 cells showed efficient internalisation of the nanoparticles and release of doxorubicin to the nucleus under hypoxic conditions [181].

##### Redox-Responsive Nanoparticles

Oxidative stress is usually produced by the accumulation of reactive oxygen species (ROS). There is a balance between ROS and antioxidants (redox homeostasis) in normal cells, while increased ROS levels in cancer cells reflect genomic instability, activated oncogenic signalling and metastatic potential. Cancer cells can adapt to a wide range of environmental conditions, such as persistent oxidative stress, by shifting their redox environment to a more reductive condition and overexpressing antioxidant enzymes, leading to the upregulation of several redox-sensitive pro-survival pathways. Redox-sensitive nanoparticles are designed to achieve triggered controlled release of loaded therapeutic drugs in response to intracellular redox potential. These stimuli-responsive drug delivery systems usually incorporate a disulphide linkage and are, therefore, prone to degradation by intracellular reducing molecules, primarily Glutathione (GSH). Tumour tissues exhibit a high concentration of GSH in the cytoplasm compared to healthy tissues [182]. Li et al. (2012) formulated hyaluronic acid-deoxycholic acid (HA-ss-DOCA)-based micelles that contained cystamine as bioreducible linkages for the delivery of paclitaxel. The rate of paclitaxel release was higher in the presence of 20 mM GSH when compared to the absence of GSH, as revealed through the in vitro drug release study. The cytotoxic effect in MDA-MB-231 cell lines was superior to the commercial drug Taxol, and biodistribution studies exhibited improved nanocarrier accumulation in the tumour site after 24 h. In another study, they demonstrated that these stimuli-responsive nanocarriers were rapidly and efficiently internalized in MDA-MB-231 cells by CD44 receptor-mediated endocytosis, and showed more efficient tumour inhibition compared to Taxol [183]. A different study reported the development of redox-sensitive folic acid conjugated with biodegradable polyether ester copolymer nanoparticles for the concurrent delivery of doxorubicin and indocyanine green (imaging and hyperthermia agent) for combined chemotherapy/photothermal therapy and imaging [184].

##### Thermosensitive Nanoparticles

Thermosensitive nanoparticles are materials that can be responsive to a change in temperature in a nonlinear relationship. The function of thermosensitive (also termed ‘thermoresponsive’) drug delivery nanocarriers is to retain their loaded drug at body temperature (~37 °C) and promptly release the drug in the locally heated tumour tissue (~42 °C) corresponding to mild hyperthermia. There are several methods for inducing local hyperthermia such as focused ultrasound and electromagnetic techniques such as radiofrequency-induced hyperthermia. In the case of liposomes, the elevated temperature induces a phase transition of the lipid membrane and permeation of the encapsulated drug. For example, a thermoresponsive liposome nanocarrier loaded with doxorubicin has been evaluated using a syngeneic NDL murine breast tumour model. Complete tumour regression was elicited following cycles of liposome administration and tumour hyperthermia induced using ultrasound [185]. The use of ultrasound for the clinical delivery of liposomal doxorubicin has also been reported to be safe and feasible [186]. Hyperthermia induced by an alternating magnetic field is also being widely investigated but, in contrast, generally requires the administration of inorganic nanoparticles such as iron oxide.

##### Near-Infrared (NIR) Stimuli-Responsive Nanoparticles

Near-infrared (NIR) light has a wide “therapeutic window” from 700 nm to the micron range, which helps to provide deeper tissue penetration both for imaging and therapeutic applications. The development of cheap but powerful NIR laser diodes and light-emitting diodes has spurred on the development of tumour-specific optical imaging agents and photosensitisers combined with different targeted drug delivery systems. Moreover, the ability to detect and treat tumour tissues using NIR light is aided by differences between the optical absorption and scattering properties of healthy and diseased tissue volumes, which renders tumours more transparent. A study by Yildiz et al. reported the design and preparation of nanoparticles for NIR imaging and doxorubicin delivery to the MDA-MB-231 cells [187]. In this study, biodegradable and biocompatible polymers poly (lactic-co-glycolic acid) (PLGA) and poly (lactic acid) (PLA) were used to prepare the nanoparticles. Then, the nanoparticle surfaces were decorated with PEG and protease-cleavable polypeptide poly-l-lysine (PLL). The polypeptide was utilized as a site for coupling an NIR fluorescent dye (AlexaFluor750) for imaging with doxorubicin loaded in the hydrophobic core. The results revealed that the near-infrared fluorescence increased 33-fold, mediated by the protease-induced cleavage of the Alexa Fluor 750-labeled poly-l-lysine on the surface of the nanoparticles. The treatment of MDA-MB-231 cells significantly reduced cell viability compared to free doxorubicin.

The absorption of NIR light by tissue sensitised with NIR-absorbing nanoparticles or drugs can be exploited for therapeutic applications such as photothermal therapy (PTT), which can then be combined with chemotherapy using a nanocarrier containing a chemotherapeutic and sensitizer. The use of inorganic metallic nanoparticles for PTT has been widely studied, especially gold, owing to the strong optical absorption induced by the surface plasmon resonance. The application of PTT using organic biodegradable nanoparticles can generally be achieved by sensitising the nanoparticles with an NIR-absorbing dye such as indocyanine green (ICG). Several studies have been carried out where sensitised local hyperthermia induced by NIR irradiation has been exploited for enhancing chemotherapy drug delivery. For example, Su et al. (2015) formulated a thermosensitive polymeric nanoparticle integrating ICG, paclitaxel (PTX) and survivin siRNA into these nanoparticles as a triple-punch strategy against triple-negative breast cancer. The results showed that the prepared nanoparticles significantly enhanced the stability of ICG. The controlled release of paclitaxel in the tumour regions was elicited by the hyperthermia generated through NIR laser-irradiated ICG. Furthermore, the nanoparticles displayed antitumour efficacy owing to the combinatorial effects of chemotherapy, photothermal therapy and gene therapy, with minimal adverse effects [188].

Photodynamic therapy (PDT) offers a non-thermal way to utilise red or NIR light for inducing localised necrosis after prior administration of a photosensitizing agent [189]. The combination of PDT and chemotherapy has been investigated in several experimental breast cancer studies, but the use of PDT for enhancing chemotherapy drug delivery from nanoformulations is an emerging field. In a study by Yang et al., the release of the hydrophilic chemotherapeutic agent Tirapazamine (TPZ) from liposomes, co-loaded with an NIR-absorbing dye, IR780, was achieved following NIR laser irradiation for combination chemo-photodynamic therapy. The authors proposed that the liposomal membrane was permeabilized through oxidation processes induced by the reactive oxygen species generated through the photoexcitation of the dye [190]. Dendrimer delivery of 5-aminolevulinic acid for protoporphyrin IX-based PDT has also been investigated in breast carcinoma cells, and targeting was achieved by attaching an EGFr-homing peptide to the dendrimer [191]. The first clinical trial of PDT of primary breast cancer tumours was recently reported in patients newly diagnosed with invasive ductal breast cancer using a liposomal formulation of the hydrophobic photosensitiser verteporfin. The photosensitiser was activated by exposure to 690 nm laser light delivered via a light-diffusing fibre that was positioned within the tumour under ultrasound guidance. The results revealed photodynamic therapy-induced necrotic areas in tumour tissues following histological analysis. Moreover, the study followed up with the patients after treatment for an average of 50 months and found no adverse effects, confirming the promising role of photodynamic therapy in breast cancer management [189].

##### Multi-Stimuli Nanocarriers for Breast Cancer Therapy

Combining multiple chemical moieties that respond to different stimuli into the same nanoparticles should, in principle, improve the efficiency of the controlled release profile and targeting of nanocarriers. Based on this concept, Verma et al. fabricated dual temperature/pH-sensitive nanoparticles loaded with doxorubicin. The researchers reported that there was a greater release of doxorubicin in the presence of acidic pH and at high temperatures, and the cytotoxicity of the nanoformulation was tested in several breast cancer lines [192]. Another interesting approach that exploits the variabilities in both redox potential and pH in the tumour environment utilised a PEGylated poly (α-lipoic acid) (mPEG-PαLA) copolymer dual pH- and redox-responsive drug delivery. The amphiphilic feature of the generated mPEG-PαLA copolymer enable its self-assembly into nanoparticles in aqueous solution. mPEG-PαLA nanoparticles exhibited high drug loading efficiency (88%) for doxorubicin. Additionally, the drug-loaded nanoparticles were efficiently internalized, and subsequently released doxorubicin in 4T1 cells and exhibited improved antitumour efficacy in a 4T1 tumour-bearing mice model with reduced side effects [193]. Another study by Haijun Yu et al. reported the synthesis of pH-sensitive and NIR-responsive hybrid micelles loaded with doxorubicin. These micelles were then administered to mice bearing doxorubicin-resistant MCF-7/ADR orthotopic tumours. Under physiological conditions (pH 7.4), the micelles formed a compact nanostructure retaining doxorubicin, but released the drug in a weakly acidic intracellular environment (pH ≤ 6.2). Upon exposure to the NIR laser illumination, the hybrid micelles significantly inhibited the growth of resistant MCF-7/ADR breast tumours in mice [194].

## 5. Clinical Status of Nanomedicine for Breast Cancer Treatment

In the current clinical perspective, many of these next-generation nanoparticles have undergone clinical trials and have been approved for various indications. The clinical translation of effective nanotechnology-based inventions is rapidly evolving, and has revolutionized cancer treatment [195]. The carrier types of these anticancer nanomedicines that are presently in clinical use or undergoing clinical trials include liposomes or lipid-based nanoparticles, polymer–drug conjugates, antibody–drug conjugates, micelles, dendrimers, protein-bound nanoparticles, inorganic nanoparticles, viral vectors and cell-derived vesicles [196]. Table 5 provides a summary of the applications of organic nanoformulations in the clinical treatment of breast cancer.

### 5.1. Clinically Approved Nanoformulated Drugs

A PEGylated liposomal formulation of doxorubicin trademarked as Doxil was the first nanoformulation approved by the FDA in 1995. In a phase III clinical trial, a sample of 22,509 female patients with metastatic breast cancer were treated with either Doxil 50 mg/m^2^ or doxorubicin 60 mg/m^2^. It was shown that the overall cardiotoxicity risk was significantly reduced with Doxil (~three-fold), while both Doxil and doxorubicin were comparable regarding progression-free survival and overall survival. Doxil has been used in combination with numerous other chemotherapy drugs (such as cyclophosphamide and 5-fluorouracil, cisplatin and cyclophosphamide followed by paclitaxel) and targeted therapy such as trastuzumab for advanced cancer treatment in clinical trials, where it showed good efficacy [197]. Doxil is one of the few nanoformulations approved at present for breast cancer treatment, but there are also a number of generic equivalents of Doxil either approved or in current trials.

Myocet is a non-PEGylated liposomal formulation of doxorubicin that exhibited a comparable antitumour effect to free doxorubicin but lower cardiotoxicity than doxorubicin in patients with metastatic breast cancer [198]. Myocet (60 mg/m^2^) was administrated combined with cyclophosphamide (600 mg/m^2^) to metastatic breast cancer patients in a multicentric clinical trial, demonstrating equivalent efficacy with minimal adverse effects in comparison to free doxorubicin/cyclophosphamide combination at the same dose [199].

Caelyx is a generic example of a PEGylated liposomal formulation of doxorubicin that is approved in Europe for advanced and metastatic breast cancer. Regarding the clinical translation of polymer-based nanomedicines, Genexol^®^-PM is an example of a PEG-coated polymeric micelle formulation of paclitaxel that has been clinically approved in Europe and South Korea for the treatment of metastatic breast cancer. This nanoformulation could deliver a paclitaxel dose higher than the conventional therapy without dose-limiting toxicity, and was able to achieve a 1.8-time longer circulation half-life compared to free paclitaxel. In a phase II clinical trial, 41 patients with metastatic cancer were treated with a Genexol-PM dose of 300 mg/m^2^ over a period of 3 h every 3 weeks. Based on this clinical study, the overall response rate ranged between 43.5 and 73.7%, and the average time to progression for all patients was 9 months [200]. Another recent phase III clinical trial recorded an improved overall response rate of Genexol-PM compared to free paclitaxel treatment, with controllable toxicities in a sample of 212 patients with recurrent or metastatic HER2-negative cancer.

Abraxane is a nanoparticle albumin-bound (nab)-paclitaxel that was approved by the FDA in 2005 to treat metastatic breast cancer. Safe administration of a much higher dose of paclitaxel (2–10 mg/mL vs. 0.3–1.2 mg/mL, respectively, compared to Taxol) and shorter injection times have been achieved by Abraxane [201]. Abraxane showed remarkably enhanced efficacy of paclitaxel in clinical trials involving patients with advanced breast cancer [202]. For instance, nab-paclitaxel monotherapy exhibited a greater overall response rate (34%) compared to Taxol (19%) in a phase III clinical trial for metastatic breast cancer [203]. Furthermore, in the GeparSepto trial conducted on a sample of 1229 females with formerly untreated unilateral or bilateral primary invasive breast cancer, it was noted that substituting solvent-based paclitaxel with nab-paclitaxel resulted in a significant increase in the proportion of patients achieving a pathologic complete response rate after anthracycline-based chemotherapy, which proposes the possibility of the replacement of solvent-based paclitaxel by nab-paclitaxel for primary breast cancer treatment [204]. However, we should be cautious in interpreting improvements in progression-free survival because it often does not translate into an overall survival or even quality of life benefit.

### 5.2. Nanoformulated Drugs in Clinical Trials

Liposomal formulations of paclitaxel such as LEP-ETU and EndoTAG-1 are being investigated for metastatic breast cancer in phase II and III clinical trials, respectively. MM-302 is an example of an antibody–drug conjugate liposomal nanoformulation comprising an HER2-targeted antibody attached to doxorubicin, which is being investigated for HER2-positive, locally advanced/metastatic breast cancer in phase III clinical trials [196].

Stimuli-responsive liposome-based nanodrugs have also been investigated clinically, most commonly using thermally induced drug release, as described in Section 4 [185,186]. ThermoDox, a thermosensitive liposomal formulation of doxorubicin, was developed to release the encapsulated drug at elevated temperatures where the lipid bilayer structure becomes permeable. In the DIGNITY phase I/II study, the administration of ThermoDox in combination with mild hyperthermia was evaluated for the treatment of recurrent chest wall breast cancer in 28 patients administered with equivalent doxorubicin doses of 40 or 50 mg/m^2^. In addition to a 61.9% local response rate among patients, a combined local response rate was noticed in almost half of the cohort (46.4%), showing five durable local responses lasting more than 3 months, four complete responses and one partial response. It was also observed that patients who received the lower liposomal doxorubicin dose (40 mg/m^2^) exhibited a comparable response rate and a superior safety profile in comparison to those receiving the higher nanoformulation dose (50 mg/m^2^) [205].

NK-105 is another example of a polymeric micellar formulation of paclitaxel that is currently undergoing phase III clinical trial for the treatment of recurrent breast cancer [196]. It displayed a half-life that was more than 20 times longer than paclitaxel (longer than 10 h for NK105 and around 30 min for paclitaxel). A phase III clinical trial (NCT01644890) comparing NK105 and paclitaxel for metastatic or recurrent breast cancer showed that treatment with a dose of 65 mg/m^2^ for NK105 attained comparable efficacies to an 80 mg/m^2^ dose of paclitaxel in terms of progression-free survival (8.4 months for NK105 vs. 8.5 months for PTX), and the incidence of peripheral sensory neuropathy was 1.4% compared to 7.5% (≥Grade 3) for NK105 and paclitaxel, respectively. This indicates that NK105 was well tolerated, particularly for the peripheral sensory neuropathy profile [206].

In the clinical domain, it is evident that liposome and micelles remain the dominant nanoformulations; however, the advent of the clinical use of lipid nanoparticles (LNPs) for RNA vaccine delivery [207] and other non-cancer applications is likely to enhance their prospects for the treatment of solid cancers. Different nanomedicines are also being developed for other types of solid tumour and, in due course, may prove useful for the treatment of breast cancer. For example, liposomal irinotecan (a topoisomerase-1 inhibitor) was originally investigated clinically for the treatment of metastatic pancreatic ductal adenocarcinoma, and is now being investigated in a phase I study for metastatic breast cancer [208].

## 6. Environmental Hazards of Nanoparticles

As nanomedicine is continuously evolving, concerns have been raised regarding the safety and toxicological properties of nanomaterials and environmental hazards. As previously discussed, the long-term retention of nanoparticles within the body is of particular concern, which has led to increased attention being paid to biodegradable nanomaterials for the sake of improving the safety profile of nanomedicine. However, increasing attention is also being given to potential environmental hazards arising from the manufacture and use of nanomaterials. The widespread manufacture and utilization of nanomaterials have raised numerous concerns about their fate, life cycle and potential toxicity in the environment and to human health. The discharge of engineered nanomaterials into the environment is the final stage in their life cycle [209]. Nanomaterials could be excreted from humans in urine and faeces, and so enter the sewage system, or be released from their remains after their death, and they are eventually distributed throughout the biosphere. After being released into the environment, nanomaterials could build up in various environmental media, such as water, soil and sediments [210]. At present, the environmental exposure to nanomaterials is mostly unclear due to the lack of important information on the type, distribution and use of nanomaterials. The key factors to consider when assessing the exposure risk for nanoparticles include the form in which these materials come into contact with humans and the environment, their behaviour after release into the environment, how stable and long-lived these forms are, whether they aggregate or disintegrate, their solubility in water or body fluids, their possible interaction with other nanoparticles, chemicals and surfaces and how their properties alter throughout these processes. Unfortunately, there are very few insights available regarding the modification of nanomaterials (particularly organic nanomaterials) and changes in their physicochemical properties through environmental impacts [211]. Given that the environmental impacts of nanomaterials cannot be identified clearly and there are many variables to take into consideration, such as low detection limits and unknown environmental concentrations of the nanomaterials, it is extremely challenging to ascertain the ecological effects of nanomaterials. Even a minor alteration in the chemical structure of nanomaterials could drastically change their properties, transforming them into toxic compounds [212]. Owing to their large active surfaces, nanoparticles can bind to pollutants such as heavy metals or organic substances that are present in soil, thus posing a threat to ground water. Some nanomaterials may have a less severe effect on the environment than on human health, while others may be more hazardous for the environment. Even though a nanomaterial can be biocompatible and safe for humans, this does not mean that they are not harmful to the environment. For example, polymeric nanoparticles as reviewed herein could pose some environmental hazards. For instance, a polymeric nanoparticle may contaminate the groundwater and so be highly hazardous to crops, fish or other living species, adversely affecting the food chain and the environment [213]. Nevertheless, the availability of ecotoxicological findings varies substantially between the investigated nanomaterials. For example, in the case of chitosan, numerous ecotoxicological studies are available, in contrast to the polymers PLA, PLGA and PHA [210], although due to the biodegradability of many polymeric nanoparticles, the quantities of residues released into the environment are predicted to be markedly diminished. At present, most toxicological studies of nanomaterials address human risks rather than environmental hazards. Nevertheless, more studies are needed to provide information on any risks from environmental exposure.

## 7. Conclusions and Future Prospects

The recent advancement in molecular biology techniques and genetic sequencing analysis has improved our understanding of breast carcinogenesis and aided in the identification of various subtypes of breast cancer. This has led to substantial progress in research studies focusing on the application of nanomedicine for breast cancer diagnosis and treatment. From the literature survey of nanomedicines that are currently being evaluated, it can be observed that a myriad of nanotherapeutic approaches ranging from inorganic nanoparticles, organic nanoparticles and actively targeted nanoparticles to smart multifunctional nanocarriers are being investigated for breast cancer. This review has mainly shed light on studies that investigated the effect of biodegradable, biocompatible and biomimetic nanocarriers in breast cancer. Additionally, the ability to incorporate multiple functional moieties into the same nanodelivery system in order to enhance specific tumour targeting, the prolongation of circulation time, sustained drug release, combination therapy and tumour visualization (e.g., fluorescent dye) has revolutionized the field of nanomedicine applications in breast cancer management. Although utilizing multifunctional moieties may indeed improve the efficacy of nanocarriers, their inclusion in nano-based drug delivery systems may also lead to formulation complexity, which, in turn, may possibly lead to higher toxicity and immunogenicity, increased manufacturing cost and good manufacturing practice issues. Nevertheless, given the increasing role of combination therapy in cancer treatment, it is likely that nanodelivery systems will play an increasingly important role in the armamentarium for breast cancer treatment. Despite all these recent developments in nano-based platforms, caution must be still taken into consideration when adopting these advanced strategies for breast cancer management in clinics. Several issues should be addressed first to achieve successful translation of these nano-based platforms from bench to clinic, such as better understanding of the biological fate of nanomaterials in vivo, better appreciation of their interactions within the blood circulation and further examination of their trafficking through intracellular compartments. Nanoparticles face a long journey with many obstacles in the way to reach tumour sites. They must first evade uptake by the immune system, then bypass biological barriers in the tumour microenvironment and successfully reach their target site of action. For drugs that are borne within nanocarriers taken up via endocytosis, a key barrier to overcome is endolysosomal sequestration and the associated risk of degradation within these vesicles. Thus, developing nanoformulations that can effectively overcome all these hurdles with minimal adverse effects is of great significance, but also remains challenging. Furthermore, novel nanoformulations need to be clinically safe, sufficiently stable, cost-effective and ideally environmentally benign. Another issue that needs to be tackled is tumour heterogeneity. Given the high inter- and intra-individual heterogeneity of breast cancer, which greatly affect the therapeutic response to nanomedicines, it has become extremely important to employ biomarkers to guide patient selection and clinical trial design. These interpatient variations in breast tumour behaviour make it almost impossible to use one approach that fits all. This implies the need for tailoring a specific nanomedicine therapeutic approach for each patient. Unfortunately, at present, no biomarkers or diagnostic tests are widely available to guide the clinical translation of cancer nanomedicines. Future directions for cancer nanomedicine will involve nanobioinformatics, which brings together the science of nanotechnology with bioinformatics. Nanobioinformatics can offer new routes to finding solutions to problems involving high-throughput genomics data, complex biological systems, novel biomarker discovery and computer-aided drug design. In the longer term, such analysis could facilitate and optimise the design of drug nanocarrier systems to treat solid tumours such as breast cancer.

## Figures and Tables

**Table 1:** Lipid-based nanoparticles investigated in breast cancer.

Name of Nanoparticle	Composition/Coating of Nanodelivery System	Size	Drug/Biomolecule	Cell Line/Animal Model	Targeting	Outcome	Year	Reference
Liposomes	Phosphatidylcholine, cholesterol and DSPE-PEG2000, dichloromethane	NA	Gemcitabine	MDA-MB-231 and 4T1 cell lines/4T1 tumour-bearing mice	Passive targeting	Gemcitabine-loaded liposomes significantly inhibited cell viability and induced apoptosis compared to free drug, irrespective of cell sensitivity, both in vitro and in vivo.	2013	[30]
Liposomes	Hydrogenated phosphatidylcholine (HPC), cholesterol, Propylene glycol (PG), tween-80, 5% trehalose solution	182 nm	Epirubicin (EPI)	MDA-MB 435, (MDA-MB 435/ADR) and chemically induced tumour model	Passive targeting	Effective growth inhibition in MDA-MB-435 cells, as well as in the resistant variant MDA-MB 435/ADR cells.	2013	[31]
Liposomes	EPC/cholesterol/DSPE-PEG2000	100 nm	OCT-modified daunorubicin plus dihydroartemisinin	MCF-7 cells and MDA-MB-435S cells/DA-MB-435S cell xenografts nude mice	Active targeting of somatostatin receptors	Enhanced cytotoxicity and cellular uptake; specific tumour accumulation and antitumour efficacy.	2018	[32]
Liposomes	1,2-dioleoylsn-glycero-3-phosphoethanolamine (DOPE), cholesterol (Chol), 1,2-dimyristoyl-rac-glycero-3-methylpolyoxyethylene (DMG-PEG2000, PEG)	163 nm	Paclitaxel, camptothecin and P53 mRNA	MDA-MB-231 cell line, orthotopic TNBC model in nude mice	Passive targeting	Nanoparticles displayed synergetic cytotoxicity of paclitaxel and P53 mRNA both in vitro and in vivo.	2019	[33]
Liposomes	DSPE-mPEG2000 (distearoyl phosphoethanolamine- polyethylene glycol) and SPC (soybean phospholipids with 75% phosphatidylcholine and cholesterol.	120 nm	Cisplatin	MCF-7 cells	Passive targeting	Liposome-loaded cisplatin had greater uptake and cytotoxicity compared with cisplatin alone.	2019	[34]
Liposomes	(Soy) (HSPC), (POPC), (DOPC), (DPPC), (DSPE-PEG-2000), (Mal-PEG-2000), Cholesterol (CHO), (DOTAP) and (DDAB)	99 to 181 nm	Doxorubicin	Her-2+ MCF-7 and SKBR-3 cells	Active targeting of HER2+ by aptamer A6	Aptamer-labelled liposomes elicited higher uptake by more than 60% into Her-2+ MCF-7 and SKBR-3 cells compared to non-targeted nanoparticles.	2020	[35]
Liposomes	DPPC, CHO, GANGLIOSIDE, DSPEmPEG2000-maleimmide	142–150 nm	Doxorubicin (DOX) and sorafenib (SRF)	MCF-7 and MDA-MB-231 cell lines, 2D and 3D spheroid models	Active targeting of p32 by LinTT1 peptide	LinTT1-functionalized liposomes enhanced therapeutic efficacy of both drugs in 2D culture and in 3D spheroids of MDA-MB-231 cells.	2021	[36]
Liposomes	DOTAP, DOPE, Cholesterol, PC	200 nm	Docetaxel and SIRT1 shRNA	MCF-7, MDA-MB-231 cells and chemically induced animal breast cancer model	Passive targeting	Co-loaded NPs resulted in the highest apoptotic profile in vitro and ~52% reduction in tumour burden in animal models compared to docetaxel liposomes and free docetaxel.	2021	[37]
Liposomes	DSPE-PEG2000, SPC, CHO	127–134 nm	5-fluorouracil and paclitaxel	MDA-MB-231 cell line and tumour-bearing mouse model	Mitochondri a-targeted KLA	In vitro and in vivo studies showed that combined drug-loaded KLA-conjugated liposomes exhibited the highest apoptosis of breast cancer cell line and the highest tumour growth inhibition compared to other groups.	2022	[38]
Solid lipid nanoparticles (SLN)	Curdlan, glyceryl caprate and polyethylene glycol (PEG) 660 hydroxystearate	NA	Doxorubicin (DOX)	MCF-7 cell line and its adriamycin-resistant variant (ADR)	Passive targeting	SLN-DOX showed significant cytotoxicity as a result of doxorubicin accumulation in the cells overcoming chemoresistance.	2010	[39]
Solid lipid nanoparticles (SLN)	Hydrogenated soya phosphatidylcholine (HSPC), distearoyl phosphatidyl ethanolamine (DSPE) and cholesterol	NA	Curcumin	MCF-7 cell line	Active targeting of transferrin (Tf) receptors	Conjugated curcumin nanoparticles resulted in better delivery and enhanced cytotoxicity with active targeting against MCF-7 cells.	2010	[40]
Solid lipid nanoparticles (SLN)	Glyceryl palmitostearate, Polyoxyl 35 and Polysorbate 80	216 nm	Tamoxifen	MCF-7 cell line	Active targeting of transferrin (Tf) receptors	Tamofixen-loaded solid lipid nanoparticles induced significantly higher cytotoxicity vs. free drug	2020	[41]
Solid lipid nanoparticles (SLN)	l-α-phosphatidylcholine (PC) and DSPE–methyl (polyethylene glycol)-2000 (mPEG2,000)	NA	Paclitaxel	MCF-7 and MCF-7/ADR (ADR) cell lines	Passive targeting	Significant increase in the intracellular uptake of paclitaxel and improved anticancer activity in MCF-7/ADR cells.	2018	[42]
Solid lipid nanoparticles (SLN)	Stearylamine, Pluronic F-68	112.18 nm	Niclosamide	MDA-MB231 cell line	Passive targeting	In vitro studies of the Niclo SLNs showed better cytotoxicity than the naïve Niclo, and significant higher cell uptake after 24 h exposure.	2019	[43]
Solid lipid nanoparticles (SLN)	glycerol monostearate, soy lecithin, dichloromethane	88–114 nm	Doxorubicin	MDA-MB-468 cell line	Active targeting by anti-EGFR/CD4 4 dual-RNA aptamers	SLNs/DOX/Dexa/CD44/EGF R resulted in a significant reduction in cell viability compared to other treated groups.	2022	[44]
Solid lipid nanoparticles (SLN)	Disteroylphosphatidylet hanolamine-poly(ethylene glycol)	224–232 nm	mitoxantrone	MCF-7 breast cancer cell line	Active targeting by folic acid	Results showed high cellular uptake of folate conjugated NPs compared to untargeted NPs and improved cytotoxicity of mitoxantrone against MCF-7 cells.	2022	[45]
Nanostructured lipid carriers	Glyceryl tridecanoate, glyceryl tripalmitate, phosphocholine (NBD-PC)	32 nm	Quercetin	MCF-7 and MDA-MB-231 cell lines	Passive targeting	The solubility of Quercetin improved 1000-fold, and the cytotoxicity and apoptosis increased in a dose-dependent manner.	2014	[46]
Nanostructured lipid carriers	Cholesterol, α-tocopherol, lecithin and Poloxamer and polyethylene glycol (PEG)	154.6 nm	Paclitaxel (PTX)	MCF-7 cell line	Active targeting by folate	Cytotoxicity of paclitaxel-loaded, folic acid-PEG-modified nanoparticles was significantly enhanced compared to free paclitaxel and other drug-loaded modified nanoparticles.	2017	[47]
Nanostructured lipid carriers	PEG-SA, soybean phosphatidylcholine (S100), oleic acid, glycerin monostearate and Compritol^®^ 888 ATO	100 nm	Doxorubicin and Lapachone	MCF-7 ADR cell line/BALB/c nude mice	Passive targeting	In vitro experiments and in vivo anti-cancer assays on MCF-7 ADR mice model showed that combined drugs loaded on the nanocarrier had significant anticancer efficacy, confirming synergistic effects.	2018	[48]
Nanostructured lipid carriers	Stearic acid, oleic acid, Phospho-Lipon^®^ 90 G	82–88 nm	Resveratrol	MCF-7 breast cancer cell line	Active targeting by folate	In vitro studies showed a 2.5-fold increase in cytotoxicity of MCF-7 cells using targeted nanocarrier compared to free drug.	2019	[49]

Abbreviations: (POPC) 1-palmitoyl-2-oleoyl-glycero-3-phosphocholine; (DOPC) 1,2-dioleoyl-sn-glycero-3-phosphocholine; (Soy) (HSPC) L-α-phosphatidylcholine, hydrogenated; (DPPC) 1,2-dipalmitoyl-sn-glycero-3-phosphocholine; (ammonium salt) (DSPE-PEG-2000) 1,2-distearoyl-sn-glycero-3-phosphoethanolamine-N-[methoxy(polyethylene glycol)-2000]; (ammonium salt) (Mal-PEG-2000) 1,2-distearoyl-sn-glycero-3-phosphoethanolamine-N-[maleimide(polyethylene glycol)-2000]; (CHO) Cholesterol; (chloride salt) (DOTAP) 1,2-dioleoyl-3-trimethylammonium-propane; (DSPE) 1,2-dioctadecanoylsn-glycero-3-phosphoethanolamine; (DDAB) dimethyldidodecylammonium bromide; (SPC) Sphingosylphosphorylcholine; (NA) not available.

**Table 2:** Polymer-based nanoparticles investigated in breast cancer.

Name of Nanoparticle	Composition/Coating of Nanodelivery System	Size	Drug/Biomolecule	Cell Line/Animal Model	Targeting	Outcome	Year	Reference
Polymeric nanoparticles	Poly (lactic-co-glycolic acid)	170 nm	Doxorubicin and resveratrol (RES)	MDA-MB231/ADR and MCF-7/ADR cells and BALB/c nude mice tumour model	Passive targeting	Dual drug-loaded nanoparticles exhibited significant cytotoxicity on cells. In mice, nanoparticles delivered both drugs mainly to tumours with significantly inhibited growth compared with free doxorubicin.	2016	[50]
Polymeric nanoparticles	Chitosan	<150 nm	Docetaxel and cMET siRNA	Mucin1+ SK-BR-3 vs. mucin1CHO cells	Active targeting by mucin1 aptamer	Higher cellular uptake of aptamer-conjugated nanoparticles in cells overexpressing mucin1. cMET gene silencing was confirmed by significantly reduced expression of the genes involved in tumourigenicity, metastasis and angiogenesis.	2018	[51]
Polymeric nanoparticles	Poly (Cyclohexene Phthalate)	100 nm	Dasatinib	MDA-MB-231 and BT549 cell lines	Passive targeting	Dasatinib-loaded polymeric nanoparticles showed higher efficacy compared to free drug.	2019	[52]
Polymeric nanoparticles	Polybutyleneadipate-co-butylene terephthalate	NA	Docetaxel	BT-474 (HER-2-positive) and MDA-B-468 (HER-2-negative) cell lines	Active targeting by HER-2 aptamer	Significantly higher uptake in BT-474 cells compared to MDA-B-468 and higher cytotoxicity compared to free docetaxel. Lower relative migration suggested improved inhibition of migration by nanoparticles.	2019	[53]
Polymeric nanoparticles	Poly-lactic-co-glycolic acid (PLGA-PEG)	107 nm	Methotrexate (MTX) and trapoxin (TPX)	MCF-7 cell line	Passive targeting	TPX/MTX-co-loaded PEG-PLGA nanoparticles caused a significant dose-dependent decrease in cell viability with synergistic antitumour effects and activation of the mitochondrial apoptosis pathway.	2021	[54]
Polymeric nanoparticles	polyethylene glycol-polycaprolactone (PEG-PCL)	106–152 nm	Gemcitabine and MUC1 inhibitor	MCF-7 and MDAMB-231 cell lines, Ehrlich ascites carcinoma (EAC) tumour-bearing animal model	Active targeting	Gem-MUC1 Inhibitor NPs showed sustained drug release in vitro, and tumour-targeting ability with enhanced effectiveness of anticancer drugs in vitro and in vivo.	2022	[55]
Polymeric nanoparticles	PLGA-PEG	98.1 nm	PD-L1 siRNA	MDA-MB-231, BT-549 and BT474 cell lines	Active targeting by sTN145 RNA aptamer	In vitro findings revealed specific uptake of aptamer-linked NPs by TNBC MDA-MB-231 and BT-549 cells with almost complete suppression of PD-L1 expression.	2022	[56]
Polymeric micelles	MPEG2000-PDLLA2000	22.83–25.8 nm	Paclitaxel and Lapatinib (LP)	SKBr-3 (HER-2-positive) and MDA-MB-231 (HER-2-negative) cell lines	Active targeting of HER2	LP co-loaded polymeric micelles showed significantly greater cytotoxicity to SKBr-3 cells and almost no significantly different cytotoxicity to MDA-MB-231 cells compared to paclitaxel-loaded micelles.	2015	[57]
Polymeric micelles	Poly (ethylene glycol)-bock-poly(lactide) (PEG2k-PLA5k)	102.5–110 nm	Doxorubicin and curcumin	MCF-7 and MCF7/ADR cell lines/BALB/c nude mice bearing MCF7/ADR tumours	Passive targeting	In vitro studies showed that co-loaded micelles were superior to free doxorubicin, free combination (doxorubicin curcumin) and doxorubicin-loaded micelles in the inhibition of proliferation of resistant cells. Dual-loaded micelles showed enhanced tumour accumulation and strong tumour growth inhibition.	2016	[58]
Polymeric micelles	Monomethoxy poly (ethylene glycol)poly(ε-caprolactone) (mPEG-PCL) and monomethoxy poly (ethylene glycol)-poly (D, L-lactic acid) (mPEG-PLA)	24–26 nm	Docetaxel	4T1 cells and MCF-7 cell lines/4T1 tumour model in mice	Passive targeting	Encapsulation of docetaxel in micelles enhanced its water solubility with high encapsulation efficiency and cytotoxicity on 4T1 and MCF-7 cells in vitro. Anti-cancer activity on 4T1 tumour model in vivo suggested the excellent efficacy of DTX micelles.	2017	[59]
Polymeric micelles	β-cyclodextrin-{poly(ε-caprolactone)poly(2-aminoethylmethacrylate)}21	150 nm	Camptothecin	MCF-7 and 4T1 cell lines/4T1 tumour model in mice	Active targeting by nucleolin aptamer (AS1411)	Aptamer-functionalized NPs showed higher internalization and cytotoxic effects in cancer cells compared to normal cells in vitro. In vivo experiments demonstrated significant tumour growth inhibition.	2022	[60]
Polymeric micelles	b-poly(ε-caprolactone), tocopheryl polyethylene glycol succinate (TPGS)	117–137 nm	Curcumin	4T1 cell lines/4T1 tumour model in mice	Active targeting of CD44 by hyaluronan	In vivo experiments showed high tumour uptake and antitumour efficacy.	2023	[61]
Dendrimers	Pluronic F68 (PF68)-conjugated polyamidoamine (PAMAM)	200–400 nm	Doxorubicin	MCF-7/ADR cell line, MCF-7/ADR tumour spheroids and MCF-7/ADR tumour-bearing nude mice MCF-7 cell line	Passive targeting	Increased antitumour activity of the doxorubicin-loaded dendrimers was demonstrated in vitro and in vivo.	2016	[62]
Dendrimers	Hyperbranched polyglycerol derivative (HPG-C18) and dendritic poly(L-lysine) (PLLD)	100 nm	Docetaxel and MMP-9 siRNA	MCF-7 murine tumour model	Passive targeting	In vitro studies showed that the combined drug-loaded dendrimers caused significant apoptosis compared to docetaxel or MMP-9 alone, and stronger tumour inhibition.	2016	[63]
Dendrimers	Pluronic F68 (PF68)-conjugated polyamidoamine (PAMAM)	NA	Doxorubicin	T47D cell line	Active targeting by antiCXCR4 antibody, which binds to CXCR4 receptors on cancer cells	AntiCXCR4 doxorubicin-loaded dendrimers induced a significant increase in in vitro cytotoxicity compared to non-targeted dendrimers. They also showed a remarkable reduction in the migration of BT-549-Luc breast cancer cells.	2017	[64]
Dendrimers	Amine terminated pluronic F68 (PF68)-conjugated polyamidoamine (PAMAM) G4	NA	Docetaxel/Paclitaxel	MCF-7 (HER-2-negative) cell line and SKBR-3 (HER-2-positive) cell line	Active targeting of HER2 by trastuzumab	Drug-loaded trastuzumab-conjugated PAMAM dendrimers exhibited remarkably high toxicity on SKBR-3 cells and very low toxicity on MCF-7 cells.	2019	[65]
Dendrimers	polyamidoamine (PAMAM) G4	113.3–206.7 nm	Methotrexate and high-mobility group protein A2 (HMGA2) siRNA	MCF-7 and MDAMB231 cancer cell lines	Active targeting of folate receptor	G4/MTX-siRNA showed strong internalization, resulting in significant apoptosis-mediated cell death by specific downregulation of HMGA2 expression.	2021	[66]
Dendrimers	poly-lysine dendrimers	180 nm	Doxorubicin and P-gp siRNA, Bcl-2 siRNA	MCF-7 and MCF7/ADR cancer cell lines and orthotopic breast tumour model in mice	Active targeting of cells overexpressing sialic acid by phenylboronic acid	In vitro studies demonstrated that targeted NPs were capable of suppressing the proliferation of MCF-7/ADR cancer cells, and in vivo studies revealed significant tumour growth inhibition.	2022	[67]
Lipid–polymer hybrid nanoparticles	Carboxylic modified latex (CML) polystyrene nanoparticles, phospholipid functionalized liposomes composed of DSPC:Cholesterol:POPG	120 nm	Doxorubicin and siRNA for multidrug resistance protein 1 (MDR-1)	MDA-MB-468 cell line/MDA-MB468 cells xenograft animal model	Active targeting of CD44 receptors by hyaluronic acid	A single dose significantly lowered the expression of MDR-1 in tumours by almost 80%. Combining siRNA resulted in a four-fold improvement of the efficacy of doxorubicin in vitro and up to an eight-fold reduction in tumour volume compared to the control treatments.	2013	[68]
Lipid–polymer hybrid nanoparticles	(DSPE-PEG 2000), (DSPE), NBD-PE c) Poly(lactide-co-glycolide) (PLGA)	230 nm	Docetaxel (DTX)	SK-BR-3 cells (Her2-positive cell line) and MDAMB-435	Active targeting by antiHER2/neu peptide (AHNP) and modified HIV-1 Tat (mTAT) for cell membrane penetration	Docetaxel-loaded dual ligand hybrid nanoparticles exhibited gradual sustained drug release. They were also substantially more potent against SK-BR-3 cancer cells than other nanoparticle formulations and free drug.	2015	[69]
Lipid–polymer hybrid nanoparticles	PLA, DSPE-PEG, SA (stearyl amine)	71 nm	Methotrexate and beta carotene	MCF-7 cell line/tumour model in rats	Active targeting by fructose	Fructose-methotrexate-beta carotene-loaded nanoparticles had the highest cytotoxic effect against MCF-7 cells and the greatest tumour growth suppression in the animal model.	2017	[70]
Lipid–polymer hybrid nanoparticles (LPHNP)	DSPE-PEG (2000)NH2, PLGA	143 nm	Docetaxel (DTX)	MDA-MB-231 cell line/MDA-MB231 cells tumour model in mice	Passive targeting	Enhanced cytotoxicity and greater cellular uptake of docetaxel in breast cancer cells, and better in vivo pharmacokinetic profile.	2019	[71]
Lipid–polymer hybrid nanoparticles (LPHNP)	SPC, DSPE-PEG2000, PLGA	~200 nm	Gemcitabine	MCF-7 and MDAMB-231 cell lines/NMU-induced breast tumour model in rats	Passive targeting	GEM-loaded LPHNs significantly reduced cell viability of both cancer cell lines in vitro and showed enhanced antitumour efficacy in rats.	2020	[72]
Lipid–polymer hybrid nanoparticles (LPHNP)	Dimethyldioctadecylammonium bromidemethoxy poly (ethylene glycol)-poly (εcaprolactone)	80–90 nm	Insulin-like growth factor type I (IGF-1R) siRNA	MCF-7 human breast cancer cell line	Passive targeting	IGF-1RsiRNA-loaded LPHNs caused significant cytotoxicity of MCF-7 cells with a significant decrease in IGF-1R mRNA expression compared to free IGF-1RsiRNA.	2021	[73]
Lipid–polymer hybrid nanoparticles (LPHNP)	Lipoid-90H and chitosan	218–439 nm	Sunitinib	MCF-7 breast cancer cell line	Passive targeting	In vitro studies showed potent cytotoxicity against MCF-7 breast cancer cells.	2022	[74]

Abbreviations: (PDLLA2000) Poly-d,l-lactic acid 2000, (MPEG2000) methoxy(polyethylene glycol) 2000, (PEG2k-PLA5k) polyethylene glycol-Poly lactic acid, (DSPC) Distearoylphosphatidylcholine, 1-(POPG) Palmitoyl-2-oleoyl-sn-glycero-3-(phospho-rac-(1-glycerol), (DSPE-PEG-2000) 1,2-distearoyl-sn-glycero-3-phosphoethanolamine-N-[methoxy(polyethylene glycol)-2000], (NBD-PE c) 7-nitro-2-1,3-benzoxadiazol-4-yl; (PLGA) Poly(lactide-co-glycolide).

**Table 5:** Nanoparticles clinically approved or under clinical trial investigation for breast cancer.

Name of Product	Type of Nanoparticle	Drug/Biomolecule	Specific Indication and Confirmed Benefit	Approval/Clinical trial Status
EndoTAG-1	Liposome	Paclitaxel	HER2-negative relapsed or metastatic TNBC	Phase III
LEP-ETU (Liposomal Entrapped Paclitaxel-Easy To Use)	Liposome	Paclitaxel	Metastatic breast cancer	Phase II
LIPUSU^®^	Liposome	Paclitaxel	Metastatic breast cancer	Phase IV
nal-IRI	Liposome	Irinotecan	Metastatic breast cancer	Phase I
Doxil^®^ (US) Caelyx^®^ (Europe)	PEGylated Liposome	Doxorubicin hydrochloride	Metastatic and advanced breast cancer—reduced cardiac toxicity	Approved
Myocet	Non-PEGylated Liposome	Doxorubicin citrate	Metastatic breast cancer Reduced cardiac toxicity	Approved in Europe and Canada/Phase III US
MM-302	HER2-targetingLiposome	Doxorubicin	HER2-positive, locally advanced/metastatic BC	Phase III
2B3-101	GSH PEG-liposome	Doxorubicin	Metastatic	Phase II
Lipolatin (regulon Inc.)	PEGylated liposome	Cisplatin	Metastatic	Phase III
Mitoxantrone HCL Liposome	Liposome	Mitoxantrone	Advanced recurrent/metastatic breast cancer	Phase II
ThermoDox^®^	Heat-activated liposome	Doxorubicin	Refractory chest wall breast cancer	Phase I/II
Narekt-102	PEGylated liposome	Irinotecan	Metastatic	Phase III
SPI-077	Stealth liposomal	Cisplatin	Advanced breast cancer	Phase I/II
DEP^®^ docetaxel	PEGylated dendrimer	Docetaxel	Locally advanced or metastatic breast cancer	Phase II
NK-105	mPEG-b-poly (asparticacid) micelles	Paclitaxel	Metastatic or recurrent breast cancer	Phase III
Nanoxel M^®^	PEG-poly (D, L-lactide)	Docetaxel	Metastatic	Phase I
Genexol^®^-PM	Polymeric micelle PEG-poly (D, L-lactide)	Paclitaxel	Metastatic breast cancer. Improved progression-free survival only and not overall survival	Phase III US/(Approved in Europe and South Korea)
Abraxane/Nab-paclitaxel	Human serum albumin	Paclitaxel	Metastatic breast cancer. Improved progression-free survival only and not overall survival	Approved by US FDA and EuropeanMedicine Agency in 2005
ABI-008	Human serum albumin	Docetaxel	Metastatic breast cancer	Phase II
